# Wire-Shaped 3D-Hybrid Supercapacitors as Substitutes for Batteries

**DOI:** 10.1007/s40820-019-0356-z

**Published:** 2020-01-20

**Authors:** Kyeong-Nam Kang, Ananthakumar Ramadoss, Jin-Wook Min, Jong-Chul Yoon, Deokjung Lee, Seok Ju Kang, Ji-Hyun Jang

**Affiliations:** 1 School of Energy and Chemical Engineering, Low Dimensional Carbon Materials Center, Ulsan National Institute of Science and Technology, Ulsan, 44919 Republic of Korea; 2 Laboratory for Advanced Research in Polymeric Materials, Central Institute of Plastic Engineering and Technology, Bhubaneswar, 751024 India; 3 School of Mechanical, Aerospace and Nuclear Engineering, Ulsan National Institute of Science and Technology, Ulsan, 44919 Republic of Korea

**Keywords:** Three-dimensional (3D) metal current collector, Flexible hybrid supercapacitor, Wire-shaped supercapacitor, High energy density device, Fast charging energy storage system

## Abstract

We report a wire-shaped three-dimensional (3D)-hybrid supercapacitor with high volumetric capacitance and high energy density due to an interconnected 3D-configuration of the electrode allowing for large number of electrochemical active sites, easy access of electrolyte ions, and facile charge transport for flexible wearable applications. The interconnected and compact electrode delivers a high volumetric capacitance (gravimetric capacitance) of 73 F cm^−3^ (2446 F g^−1^), excellent rate capability, and cycle stability. The 3D-nickel cobalt-layered double hydroxide onto 3D-nickel wire (NiCo LDH/3D-Ni)//the 3D-manganese oxide onto 3D-nickel wire (Mn_3_O_4_/3D-Ni) hybrid supercapacitor exhibits energy density of 153.3 Wh kg^−1^ and power density of 8810 W kg^−1^. The red light-emitting diode powered by the as-prepared hybrid supercapacitor can operate for 80 min after being charged for tens of seconds and exhibit excellent electrochemical stability under various deformation conditions. The results verify that such wire-shaped 3D-hybrid supercapacitors are promising alternatives for batteries with long charge–discharge times, for smart wearable and implantable devices.

**Electronic supplementary material:**

The online version of this article (10.1007/s40820-019-0356-z) contains supplementary material, which is available to authorized users.

## Highlights

- The flexible 3D porous structure with a large surface area provides pathways for rapid ion/electron transport and ion diffusion as well as numerous electroactive sites.
- The wire-shaped supercapacitor exhibits a high energy density of 153.3 Wh kg^−1^ and a power density of 8810 W kg^−1^.
- The hybrid device demonstrates excellent durability under various mechanical deformations.

**Electronic supplementary material:**

The online version of this article (10.1007/s40820-019-0356-z) contains supplementary material, which is available to authorized users.

## Graphical abstract

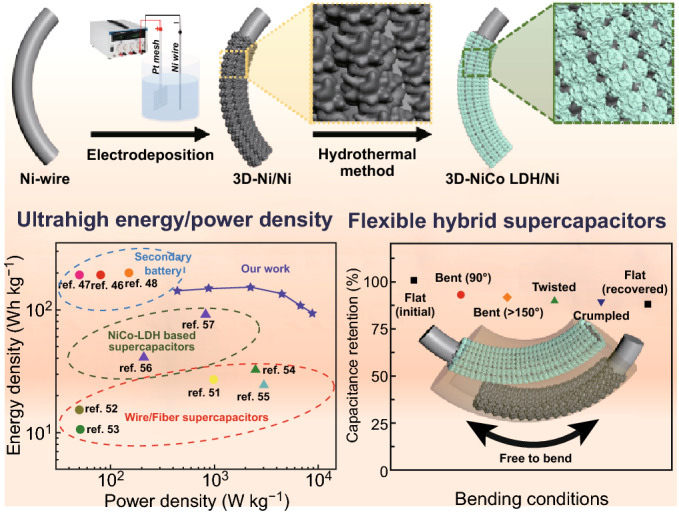

## Introduction

In recent years, smart electronics have drawn significant attention for prospective applications in wearable devices [1–5]. In line with this, there is intense demand for reliable power sources as well as high-performance, flexible, light-weight, and safe energy conversion and storage systems [6–10]. Supercapacitors are considered one of the most important energy storage devices due to their features of high power delivery ability, fast charge/discharge rates, and long cycle life [11–14]. To practically apply flexible and wearable supercapacitors, high volumetric capacitance and excellent energy density are important factors. However, a highly compact electrode structure in a solid electrolyte, essential for wearable applications, is expected to hinder ion accessibility to the electrode surface. Indeed, it has been reported that both restricted ion transport and limited faradic charge transfer process in solid compact electrodes are major contributors to the reduced capacitance (capacity) and poor rate capability of the battery-type supercapacitors [15, 16]. It is a good strategy to utilize nanostructured battery-type materials such as metallic-layered double hydroxides deposited onto flexible to ensure high surface exposure of the electrode into electrolytes for high-performance wearable supercapacitors. Taking this into account, it is critical to consider the balance between volumetric/areal capacitance, which is related to space saving, and high energy density, related to practical usage, when fabricating capacitive materials for smart wearable electronic devices. Several advanced materials such as two-dimensional (2D) metal carbides and nitrides (MXene) charged by the intercalation mechanism [17], g-C_3_N_4_ charged by the electric double-layer formation and pseudocapacitive behaviors [18], and LDHs charged by faradaic reactions have been suggested to deliver both high energy density and excellent capacitance/capacity. Among them, we chose nickel and cobalt-based LDHs with a high theoretical capacitance/capacity due to their flexible ion exchange property, good redox activity, low cost, and environmentally friendly nature.

Herein, we demonstrate the fabrication of a wire-type hybrid supercapacitor with both high volumetric capacitance and high energy density. This supercapacitor is composed of three-dimensional (3D)-nanostructured nickel wires coated in a 3D nickel cobalt-layered double hydroxide (NiCo LDH/3D-Ni) as an active material and polyvinyl alcohol–potassium hydroxide (PVA–KOH) gel as a polymer electrolyte.

The as-prepared electrode holds a structure comprising abundant 3D-interconnected porous dendritic walls for easy access of electrolyte ions and highly conductive networks for fast electron transfer and provides numerous electroactive sites for improved charge storage. The 3D-Ni fiber bends freely and acts as a binder and conductive additive-free current collector, and it also provides additional faradaic energy storage. Thus, the hybrid supercapacitors delivered excellent electrochemical capacitive properties (fast charging) and maintained outstanding electrochemical performance reliability under various deformation conditions.

## Experimental Section

### Materials

The 0.5-mm-diameter nickel (Ni) wire was obtained from Nilaco, Japan. The nickel chloride (NiCl_2_), ammonium chloride (NH_4_Cl), nickel nitrate (Ni(NO_3_)_2_·6H_2_O), cobalt nitrate (Co(NO_3_)_2_·6H_2_O), hexamethylene tetraamine (HMTA), and polyvinyl alcohol (PVA) were purchased from Sigma-Aldrich, Korea. Potassium hydroxide (KOH) was purchased from Samchun Pure Chemical Co., Ltd. Korea. All the chemicals used are of analytical grade purity and used without any further purification. Ultrapure (De-ionized, DI) water was used for all experiments.

### Preparation of 3D-Ni Film on Ni wire (3D-Ni/Ni)

Nickel wire (0.5 mm) was used as a substrate. The 3D porous nickel film on Ni wire (5 cm in length) (3D-Ni/Ni) was deposited using electrodeposition with a hydrogen bubble template method [1, 2]. The Ni wire and platinum mesh were used as the cathode and anode, respectively, for constructing the 3D-Ni film, keeping the distance between the two electrodes at 1 cm. The 3D-Ni/Ni was electrodeposited at a constant current of 2.5 A using a regulated DC power supply with the electrolyte containing 0.1 M NiCl_2_ and 2 M NH_4_Cl. After the deposition, the 3D porous Ni film was rinsed several times in DI water and dried at 60 °C for 12 h in a hot air oven.

### Preparation of NiCo LDH/3D-Ni Nanosheets Arrays

The bimetallic double hydroxide nanostructure was prepared onto the 3D Ni/Ni by a hydrothermal method. First, 0.145 g of Ni(NO_3_)_2_·6H_2_O, 0.291 g of Co(NO_3_)_2_·6H_2_O, and hexamethylene tetraamine (HMTA, 5 mmol) were dissolved in 20 mL of ethanol and 20 mL of DI water at room temperature to form a pink solution. Subsequently, the obtained solution and 3D-Ni/Ni were transferred into a 100 mL bottle with a blue cap and heated to 80 °C in an oil bath for 8 h to obtain NiCo LDH/3D-Ni nanosheets. After being cooled to room temperature, the obtained samples were washed with DI water and ethanol several times and dried at 60 °C for 12 h. The mass loadings of NiCo LDH/3D-Ni were calculated to be 0.29 mg.

### Preparation of Mn_3_O_4_/3D-Ni Nanosheet Arrays

A Mn_3_O_4_ nanosheet arrays were prepared onto the 3D-Ni/Ni by the electrodeposition method. First, 100 mM manganese acetate tetrahydrate (Mn(CH_3_COO)_2_·4H_2_O) and 100 mM sodium sulfate anhydrate (Na_2_SO_4_) were dissolved in 100 mL of DI water at room temperature. Continuously, the Mn_3_O_4_ nanosheets were electrodeposited on the 3D-Ni/Ni at 5 mA cm^−2^ for 5 min at 25 °C. The deposited electrodes were carefully washed with DI water and heat treatment was performed at 200 °C for 3 h. The mass loading of Mn_3_O_4_/3D-Ni was calculated to be 0.6 mg.

### Fabrication of Flexible All-Solid-State Hybrid Wire Supercapacitors

For a flexible solid-state hybrid supercapacitor, a PVA/KOH gel electrolyte was first prepared by the following method. First, 10 g of PVA was dissolved in 100 mL of DI water at 95 °C under stirring until the solution became clear. Then, 5.6 g of KOH was added to the above solution, which was vigorously stirred at 95 °C until a clear gel was formed. We chose PVA/KOH as an efficient polymer electrolyte because (1) PVA as a host polymer is known to provide the highest current operation of the electrode compared to any other host polymer sources because PVA is a linear polymer which forms polymeric networks among the various kinds of host polymers for hydrogel electrolytes and it has high ionic conductivity. (2) Electroactive materials are more stable and active in alkaline-based electrolytes (KOH). (3) The solidification of the electrolyte in energy devices avoids the risk of electrolyte leakage from the cell and makes it possible to fabricate devices for flexible/wearable application [19]. The two electrodes were then closely and parallel assembled onto a PET substrate with a separation distance of ~ 1 mm to form an all-solid-state flexible hybrid wire supercapacitor and again dried at room temperature overnight to remove excess water in the electrolyte. After the PVA/KOH gel electrolyte solidified, the solid-state hybrid wire supercapacitor was sealed with tape to prevent absorption of moisture.

### Material Characterizations

The morphologies and elemental composition of the as-prepared samples were characterized using a field emission scanning electron microscopy (FE-SEM, Hitachi, S-4800) equipped with energy-dispersive X-ray spectroscopy (EDS). The X-ray diffraction (XRD) patterns of the samples were examined with the 2θ-angle from 5° to 80° on a Rigaku D/max 2550 diffractometer, using Cu (Kα) radiation (*λ* = 1.5406 Å). Raman spectra were recorded with a Raman spectroscopy (WITec). The compositional analysis of the samples was analyzed by X-ray photoelectron spectroscopy (XPS, K-alpha; Escalab 250Xi model, Thermo Fisher, UK).

### Electrochemical Characterization

All electrochemical tests including cyclic voltammetry, galvanostatic charge/discharge, and electrochemical impedance spectroscopy (EIS) were performed using a three-electrode system in 2 M KOH with electrochemical workstations [VMP3 biologic electrochemical workstation and VersaSTAT3 (Princeton Applied Research)]. The as-prepared samples, platinum mesh, and Hg/HgO were used as working, counter, and reference electrodes, respectively. Mass loading of the whole electrodes and active material were measured by XS105 DualRange (METTLER TOLEDO). Further, a hybrid solid-state wire supercapacitor using the NiCo LDH/3D-Ni electrode (positive) and Mn_3_O_4_/3D-Ni (negative electrode) were tested in a two-electrode configuration using PVA–KOH gel electrolyte. Detailed information about the calculations of specific capacitance, energy density, and power density is given in the Supporting Information (SI).

### Calculation Methods

The optimum mass ratio of positive electrode to negative electrode is calculated by Eq. 1:

1 $$m_{ + } /m_{ - } = V_{ - } C_{ - } /V_{ + } C_{ + }$$

where *m* is the mass of electroactive materials, *V* is the potential window, and *C* represents the specific capacitance.

The gravimetric, volumetric, areal, and length capacitance of NiCo LDH/3D-Ni nanostructures electrode materials were estimated from the cyclic voltammetry and galvanostatic charge/discharge profiles using Eqs. 2–7.

Cyclic voltammetry:

Capacitance:

2 $$C = \frac{{\int {i{\text{d}}V} }}{2S\Delta V}$$

Galvanostatic charge/discharge:

Capacitance:

3 $$C = \frac{I \times \Delta t}{\Delta V}$$

Gravimetric capacitance:

4 $$C_{\text{g}} = \frac{c}{m}$$

Volumetric capacitance:

5 $$C_{\text{v}} = \frac{c}{v}$$

Areal capacitance:

6 $$C_{\text{a}} = \frac{c}{a}$$

Length capacitance:

7 $$C_{\text{l}} = \frac{c}{l}$$

where *C* is the capacitance (F), *C*_g_ is the gravimetric capacitance (F g^−1^), *C*_v_ is the volumetric capacitance (F cm^−3^), *C*_a_ is the areal capacitance (F cm^−2^), *C*_l_ is the length capacitance (F cm^−1^), *S* is the sweep rate (mV s^−1^), ∆*V* is the potential window (V), *I* is the discharge current (A), ∆*t* is the discharge time (s), *v* is the volume of the electrode material (cm^3^), *m* is the mass of the active material (g), *A* is the area of the electroactive material (cm^2^), *l* is the length of the electrode material, and ∫*i*d*V* is the integral area of the CV curve (A).

8 $$E = \frac{1}{2}C_{\text{t}} V^{2}$$

9 $$P = \frac{E}{t}$$

where *C*_t_ is the specific capacitance of the supercapacitor based on the total mass of the two electrodes (F g^−1^), *V* is the potential window in the discharge process, and *t* is the discharge time (s).

## Results and Discussion

### Characterization of NiCo LDH/3D-Ni Electrode

**Fig. 1:**
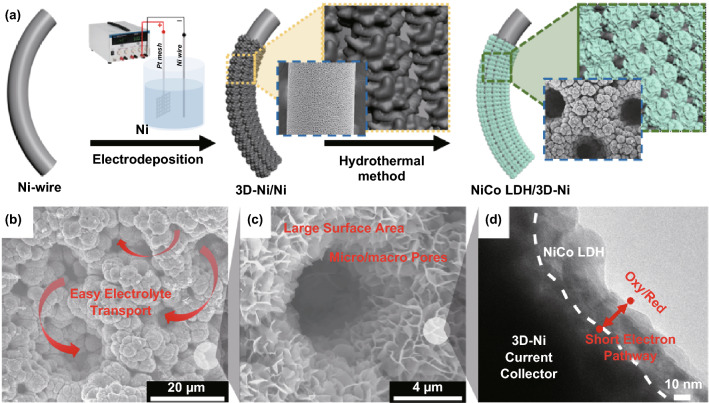
**a** Schematic representation of the fabrication of the NiCo LDH/3D-Ni electrode. **b, c** FE-SEM images, and **d** TEM image of the NiCo LDH/3D-Ni nanostructures at different magnifications

The fabrication procedure of the NiCo LDH/3D-Ni nanostructure is schematically presented in Fig. 1a. First, 3D-Ni porous structures were constructed onto Ni wire substrates via electrodeposition using a hydrogen bubble template method. The 3D porous interconnected Ni dendritic walls over the Ni wire electrode (3D-Ni/Ni) are shown in Fig. S1. The highly porous, conductive, and large surface area 3D-Ni metal is essential for the growth of various nanostructures in small areas and therefore efficiently serves as a cost-effective and compact electrode for hybrid supercapacitors. 3D-NiCo LDH nanostructures were then grown onto the as-obtained 3D-Ni/Ni porous wire through a hydrothermal method from which NiCo LDH/3D-Ni was formed. The morphological evolution of the flexible 3D-NiCo LDH/Ni was investigated by FE-SEM and TEM (Fig. 1b–d). The 3D-Ni/Ni was homogeneously and firmly covered by the 20–30 nm NiCo LDH nanostructures with short electron pathways, forming a 3D highly porous dendritic flower-like structure with micron-size voids (~ 2 µm). The interconnected arrangement of the NiCo LDH nanostructures on the 3D-Ni/Ni with cavities provides excellent accessibility for electrolyte ions over the entire surface of the electrode and lower interfacial resistance, which is beneficial for abundant electrochemical reactions with gel polymer electrolyte ions in the compact electrode. A cross-sectional view of the NiCo LDH/3D-Ni nanostructure electrode at different magnifications is shown in Figs. S2, S3. XRD and XPS spectra of NiCo LDH are shown in Fig. S4.

### Electrochemical Performances of NiCo LDH/3D-Ni

**Fig. 2:**
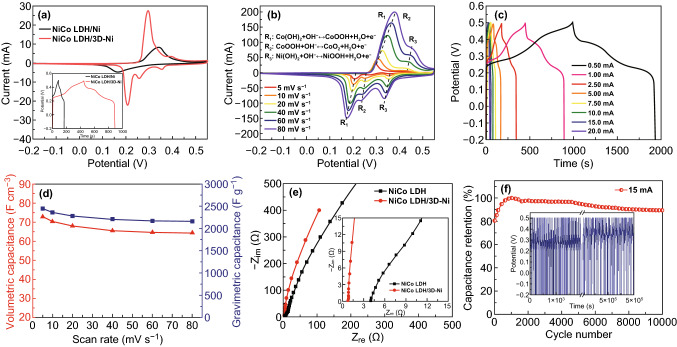
**a** Cyclic voltammetry and charge/discharge profiles of NiCo LDH/Ni, and NiCo LDH/3D-Ni nanostructures at a scan rate of 5 mV s^−1^. **b** Cyclic voltammetry profiles, **c** galvanostatic charge/discharge profiles, and **d** volumetric capacitance (specific capacitance) of NiCo LDH/3D-Ni nanostructures at different scan rates (ranging from 5 to 80 mV s^−1^). **e** The EIS data of the NiCo LDH/3D-Ni electrode and the NiCo LDH/Ni electrode. **f** Cyclic stability obtained at a current of 15 mA (current density: 51.72 A g^−1^); the inset shows the charge/discharge curves

The electrochemical properties of the NiCo LDH/3D-Ni nanostructures were investigated as electrode materials for hybrid supercapacitors. Figure 2a shows CV curves of NiCo LDH/Ni and NiCo LDH/3D-Ni at a scan rate of 5 mV s^−1^. It can be observed that the NiCo LDH/3D-Ni exhibited a larger CV integrated area than NiCo LDH nanosheets on the flat surface, signifying that NiCo LDH active materials afford a much higher electrochemical performance with effective charge transfer when in the form of a 3D-configuration in Fig. S5. The potential difference between the anodic peak (*I*_pa_) and the cathodic peak (*I*_pc_) of NiCo LDH/3D-Ni is much lower than that of NiCo LDH/Ni, indicating more reversible reactions and better kinetic properties in NiCo LDH/3D-Ni. As shown in the galvanostatic charge/discharge curves of the NiCo LDH/Ni and NiCo LDH/3D-Ni electrodes at the current of 1 mA (inset of Fig. 2a), the NiCo LDH/3D-Ni electrode exhibited higher charge/discharge times compared to that of NiCo LDH/Ni, indicating the high capacitive behavior of 3D-NiCo LDH.

The CV of NiCo LDH/3D-Ni at different scan rates, ranging from 5 to 80 mV s^−1^ is shown in Fig. 2b. The NiCo LDH/3D-Ni electrode exhibited three pairs of cathodic and anodic peaks, indicating the reversible faradaic redox process of Ni^2+^/Ni^3+^ Co^2+^/Co^3+^, and Co^3+^/Co^4+^ transitions during the CV tests. The electric energy storage mechanism for the NiCo LDH battery-type supercapacitor with three redox peaks is proposed with the equations inserted in Fig. 2b. The electrons were transported among Co^2+^, Co^3+^, and Co^4+^ ions via the proton transfer [20–26]. The charging/discharging process of Ni(OH)_2_ proceeds as the last equation insert in Fig. 2b. The phenomenon on the clear appearance of three pairs of redox peaks of NiCo LDH, compared to other previous reports with one pairs of redox peaks, can be explained by the highly porous 3D architecture which provides easy access and utilization of electrolyte ions throughout the surface of the electroactive materials, including the NiCo LDH and 3D-Ni/Ni wire at their redox potentials. Also, the CV curves show that NiCo LDH as an active material operated oxidation states changes properly in the potential window. The galvanostatic charge/discharge curve and coulombic efficiency were obtained for the NiCo LDH/3D-Ni electrode at various current densities as shown in Figs. 2c and S6. In the charge/discharge test, NiCo LDH/3D-Ni had a high discharging time due to faradaic reactions between − 0.2 and 0.5 V. However, a simple surface reaction such as formation of an electric double layer in the low potential range (0.2 to − 0.2 V) did not affect on the faradaic reaction. This tendency is consistent with the tendency of CV curves in Fig. 2b. The calculated gravimetric, volumetric, areal, and length capacitance (capacity) of NiCo LDH/3D-Ni from charge/discharge curves at a current of 0.5 mA (see SI1.2) are 2337 F g^−1^ (452.72 mAh g^−1^), 70 F cm^−3^ (13.56 mAh cm^−3^), 0.99 F cm^−2^ (0.19 mAh cm^−2^), and 0.18 F cm^−1^ (0.035 mAh cm^−1^), respectively. The obtained capacitances of NiCo LDH/3D-Ni were significantly higher than those of previously reported battery-type wire electrodes [27–32]. The volumetric capacitance values versus scan rates for NiCo LDH/3D-Ni are displayed in Fig. 2d (see Fig. S7 for more). The volumetric capacitance of the as-fabricated electrodes retained 76% of the initial capacitance, when the scan rate was increased to a high rate of 80 mV s^−1^, demonstrating good rate capability of the NiCo LDH/3D-Ni. The high volumetric capacitance values at a high scan rate compared to previous reported data [33, 34] prove that the electron/charge transport behavior was not deteriorated in the compact NiCo LDH/3D-Ni electrode due to the support of the 3D-interconnected structure, providing an excellent potential for wearable electronics. In order to systematically confirm the feasibility in the transport of electrolyte ions and electrons in the 3D-interconnected structure, EIS data of NiCo LDH/3D-Ni and NiCo LDH/Ni compared as shown in Fig. 2e. Both curves contain a semicircular arc in the high-medium frequency region, followed by a straight line in the low frequency region. In the high frequency region, the x-intercept at the beginning of the semicircle on the Z-real axis of the Nyquist plots denotes the equivalent series resistance (ESR), which comprises the solution resistance (*R*_s_), the internal resistance of the active material, and the contact resistance at the active material and substrate interface. The ESR value of the NiCo LDH/Ni and NiCo LDH/3D-Ni were obtained to be 4.1 and 0.71 Ω, respectively. The ESR value of the NiCo LDH/3D-Ni electrode was less than that of the NiCo LDH/Ni electrode, because the highly 3D porous structure facilitates transport of electrolyte ions to the surfaces of NiCo LDH deposited on the 3D-Ni/Ni current collector. In addition, it suggested the presence of tight binding between the active materials and the 3D-Ni/Ni current collectors. The semicircle in the high-medium frequency region is related to the charge transfer resistance (*R*_ct_) at the electrode/electrolyte interface. The calculated *R*_ct_ values of the NiCo LDH/3D-Ni electrode at the high-medium frequency regions were less than 0.2 Ω compared to 10 Ω for NiCo LDH, representing much faster charge transfer properties between the electrolyte and active materials in the NiCo LDH/3D-Ni electrode, which explained the outstanding kinetic properties even at high scan rates. This can be explained by the equation of *R *= *ρ* l/*A*, where the *ρ*, resistivity of the material, is dependent on the active material used. The resistance (*R*) was decreased, since the length of diffusion paths (*l*) was decreased and the active surface area (*A*) was increased by the deposition of NiCo LDH on the 3D Ni/Ni wire rather than on the Ni wire. The straight line, followed by the semicircle, in the medium frequency region is related to the diffusion of the electrolyte within the electrode. The steeper the slope becomes, the higher the diffusion capability of ions entering the pores is. The slope of the NiCo LDH/3D-Ni electrode was more vertical than that of the NiCo LDH/Ni electrode, which implied the NiCo LDH/3D-Ni was close to the ideal electrode with more facile electrolyte penetration pathways during the charge storage process. Furthermore, we evaluated diffusion coefficient as shown in Fig. S8. The calculated diffusion coefficient of NiCo LDH and 3D-NiCo LDH/Ni is 4.7624 × 10^−16^ and 2.2257 × 10^−12^ cm^2^ s^−1^, respectively, which is highly compatible with the proposed nanostructure-based morphology. The higher values of 3D-NiCo LDH/Ni specified the higher ion mobility of the electrodes due to the highly conductive porous architecture network. Aside from the superior electrochemical properties achieved by the introduction of a 3D-configuration in the compact electrode for wearable applications, the NiCo LDH/3D-Ni nanostructures further provided abundant active sites in the form of micro-/macro-size pores with a high surface area (3.52 m^2^ g^−1^) (Fig. S9). The electrochemical stability of NiCo LDH/3D-Ni was evaluated by a repeated galvanostatic charge/discharge test at a constant current of 15 mA for 10,000 cycles as shown in Fig. 2f (the inset displays the charge/discharge curves of the initial and final 50 cycles). It was observed that the specific capacitance shows a gradual increase at the first 1000 cycles and then decreases slowly until 89% of the initial capacitance remains even after 10,000 cycles, indicating good cycling stability of the NiCo LDH/3D-Ni. By continuous charge/discharge cycles and more efficient soaking time of the active materials in the electrolyte, the bulk and the interior part of the electroactive materials were activated, which lead to the increase in specific capacitance for the first 1000 cycles. The resistance characteristics of NiCo LDH/3D-Ni before and after the cycle test were further investigated by EIS measurements as shown in Fig. S10. Both EIS and FE-SEM analyses after the cycling test (Fig. S11) confirmed the structural stability of the active materials.

### Electrochemical Performances of Asymmetric Hybrid Supercapacitor

**Fig. 3:**
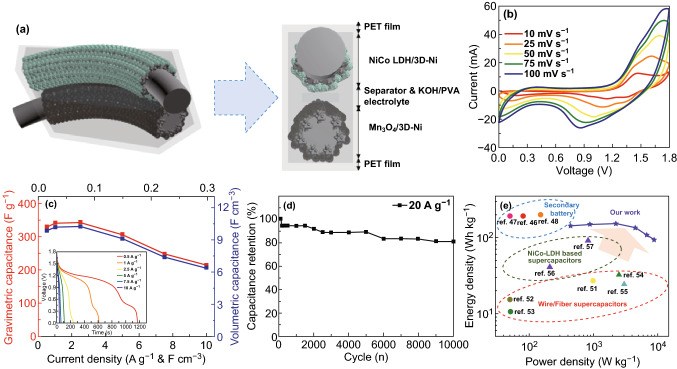
**a** Schematic representation of the fabrication of a NiCo LDH/3D-Ni//Mn_3_O_4_/3D-Ni hybrid supercapacitor. **b** CV curves of the hybrid supercapacitor at different scan rates (ranging from 10 to 100 mV s^−1^), and **c** specific capacitance of the hybrid supercapacitor at different current densities (ranging from 0.5 to 10 A g^−1^) and the inset shows the galvanostatic discharge curves. **d** Ragone plots of our supercapacitor based on a full cell compared with other electrodes using NiCo LDH and secondary batteries. **e** Cycling performance of the hybrid supercapacitor for 10,000 cycles at a current density of 20 A g^−1^

To evaluate the possibility of the as-obtained NiCo-LDH/3D-Ni wire in practical applications, a hybrid supercapacitor was fabricated by utilizing the NiCo-LDH/3D-Ni wire as the anode and the Mn_3_O_4_/3D-Ni wire as the cathode, and a schematic representation of the hybrid supercapacitor is shown in Fig. 3a. The characterization of Mn_3_O_4_ is shown in Figs. S12 and S13. The Mn_3_O_4_/3D-Ni wire electrode displayed a deformed rectangular shape at − 1.0 to − 0.2 V, as shown in Figs. S14 and S15, which specified the pseudocapacitive behavior of the electrodes. The specific capacitance of the Mn_3_O_4_/3D-Ni was calculated from its galvanostatic charge–discharge curves and reached up to 848.8 F g^−1^ at 1 A g^−1^, which is superior to any other previously reported activated carbon (AC)-based supercapacitors [35–38]. The electrochemical stability of Mn_3_O_4_/3D-Ni was evaluated by a repeated galvanostatic charge/discharge test at a constant current density of 20 A g^−1^ for 10,000 cycles as shown in Fig. S16. It was observed that over 70% of the initial capacitance was kept even after 10,000 cycles, indicating good cycling stability of the Mn_3_O_4_/3D-Ni. FE-SEM analyses after the cycling test (Fig. S17) confirmed the structural stability of the Mn_3_O_4_/3D-Ni. Before assembling positive and negative electrodes, we checked capacitive contributions of NiCo LDH/3D-Ni and Mn_3_O_4_/3D-Ni as shown in Fig. S18. Overall, the capacitive contribution increased gradually with an increase in the scan rate, whereas the diffusion contribution decreased. The ions did not have sufficient time to diffuse into the host lattices at high scan rates. Hence, the surface/near-surface behavior dominated the overall capacity. The as-fabricated hybrid supercapacitor exhibited capacitive behavior in the voltage range (0–1.8 V) with the double contribution of the electric double-layer capacitance and pseudocapacitance as shown in Fig. 3b [39]. The sum of the potential ranges of the anode and cathode electrodes in the three-electrode system must have been the same as the maximum voltage of the fiber supercapacitor in the two-electrode system if the electrolyte was the same in the both electrode systems. However, due to the low ionic mobility and conductivity of a leakage-free PVA/KOH gel polymer electrolyte in the solid phase compared to high ionic mobility and conductivity of an aqueous electrolyte, the maximum voltage of the hybrid cell (in the two-electrode system) can exceed the sum of the potential ranges of the anode and cathode electrodes in the three-electrode system. Figure 3c shows charge–discharge curves of the NiCo LDH/3D-Ni//Mn_3_O_4_/3D-Ni hybrid supercapacitor at various current densities from 0.5 to 10 A g^−1^. The specific capacitances were calculated to be 331 (162.82 mAh g^−1^), 342.5, 344.25, 307.25, 248, and 215 F g^−1^ based on the total mass of the active materials at current densities of 0.5, 1, 2.5, 5, 7.5, and 10 A g^−1^, respectively, from the galvanostatic charge–discharge curves in inset of Fig. 3c [40–45]. It was observed that the specific capacitances were high at low current densities due to activation of the hydroxide layer of hybrid electrodes and decreased at high current densities due to higher internal resistance which limits the ion transportation [46]. The calculated volumetric capacitance of the NiCo LDH/3D-Ni//Mn_3_O_4_/3D-Ni hybrid supercapacitor at a current density of 0.015 A cm^−3^ was 9.86 F cm^−3^ (4.85 mAh cm^−3^). When the current density increased, 92.8% of the volumetric capacitance was retained even at a high current density of 0.075 A cm^−3^, indicating the high rate capability of the as-prepared hybrid supercapacitor. Galvanostatic charge–discharge measurement was used to evaluate the durability of the as-fabricated hybrid supercapacitor at a current density of 20 A g^−1^ as shown in Fig. 3d. The capacitance retention after 10,000 cycles of charge/discharge was 80.7%, which implies that the hybrid supercapacitor has relatively good stability. This indicates that the hybrid supercapacitors have low electrode resistance and a high charge transfer rate between the polymer electrolyte and the active materials. The limited structural damage of the as-prepared supercapacitor with the compact assembly of the electrodes and the gel polymer electrolyte during a long charge/discharge process helped achieve superior cycling stability (Fig. S19). The Ragone plot, related to energy densities (*E*) and power densities (*P*), was further used to evaluate the performance of the NiCo LDH/3D-Ni//Mn_3_O_4_/3D-Ni device (Fig. 3e). The maximum energy density of our hybrid supercapacitor was calculated to be 153.3 Wh kg^−1^ (4.59 mWh cm^−3^) at a power density of 2238 W kg^−1^ (67.05 mW cm^−3^) based on the total mass of active materials, as shown in Fig. S20. Even at a high discharge current of 10 A g^−1^, the energy density remained at 92.8 Wh kg^−1^ (2.78 mWh cm^−3^) at a power density of 8810 W kg^−1^ (263.91 mW cm^−3^). The highest measured energy density considerably exceeded the values of almost all hybrid supercapacitors reported to date, including those based on nickel cobalt hydroxides and their composites, as well as other wire-/fiber-shape supercapacitors. The electroactive materials directly grown on the 3D-Ni network formed a better electrical connection and mechanical adhesion with the 3D-Ni current collector, which led to a reduction in the contact resistance and fast electron transport between the active materials and the 3D-Ni. The 3D porous network architecture delivers enormous open spaces for easy and rapid access of electrolyte ions (low diffusion resistance) to the innermost and outermost surfaces of the electroactive materials and rapid charge transportation (lower contact resistance) for efficient redox reactions during the faradaic charge storage process, and the large surface area of the 3D porous flower-like nanoarchitecture network provides a large density in the electroactive sites to make electrochemical reactions possible. In general, wire-shaped/fiber supercapacitors exhibit much lower energy densities because of their reduced exposed surface areas and suffer from poor mechanical performance under deformation compared to traditional planar-type supercapacitors. However, our 3D-shaped hybrid device with the numerus active sites and easy access of electrolyte ions in the solid electrolyte exhibited performance comparable to secondary batteries: Se-carbon NS//pseudographite in sodium ion battery (203 Wh kg^−1^) [46], Li_4_Ti_5_O_12_//LiCoO_2_ in Li-ion battery (200 Wh kg^−1^) [46], Co/Ni free O3-Na_0.9_[Cu_0.22_Fe_0.30_Mn_0.48_]O_2_//hard carbon in sodium ion battery (210 Wh kg^−1^) [47]. In addition to electrochemical advantages achieved by the introduction of the 3D structure in the compact wire electrode, the ultrahigh energy density also in part should benefit from the high energy contribution of the NiCo LDH and the power support of Mn_3_O_4_ directly deposited onto the 3D-Ni/Ni wire current collector [48, 49]. Also, unlike carbon-based negative electrodes with heavy mass loading to balance, the deposition of Mn_3_O_4_ lowered the amount of mass loading needed to achieve mass balance (NiCo LDH: Mn_3_O_4_ = 1:3) due to its high capacitance (848.8 F g^−1^ at 2 A g^−1^): ultrathin MnO_2_/carbon fiber (CF) (27.2 Wh kg^−1^) [50], TiN@graphene nanosheet (GNS)/CF (15.4 Wh kg^−1^) [51], CuHCF@CF (10.6 Wh kg^−1^) [52], Ni_2_CoS_4_@NiCo_2_O_4_/CFP (32.2 Wh kg^−1^) [53], Ni(OH)_2_-RGO/Ni wire (24.5 Wh kg^−1^) [54], NiCo DH/graphene/CNT composites (41 Wh kg^−1^) [55], and NiCo LDH/Ni (91.76 Wh kg^−1^) [56]. The high energy density and power density, realized in our wire-shaped 3D hybrid supercapacitors, clearly demonstrated the ability to store a lot of energy in a small volume, and to output large amounts of energy based on its volume and therefore is suitable for practical flexible applications for alternatives to batteries.

### Practical Demonstrations of the Hybrid Device

**Fig. 4:**
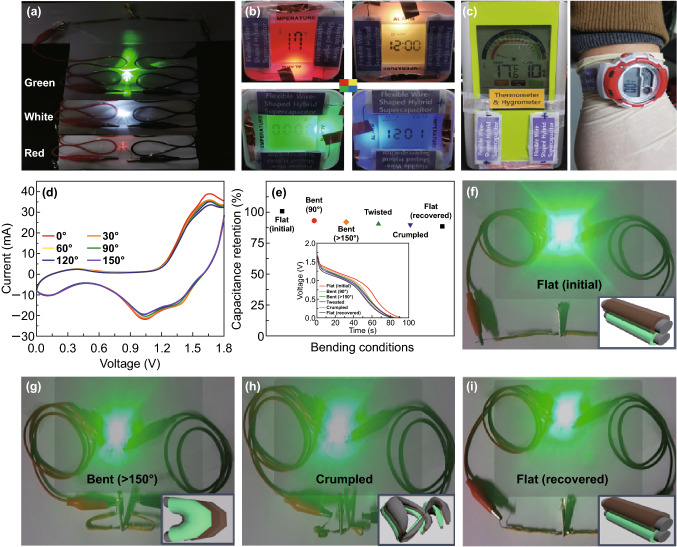
**a**–**c** Various examples of electronic devices powered by two wire-type hybrid supercapacitors in series: a digital image of **a** the green, white, and red LED indicators, **b** LCD devices with various colors, and **c** a thermometer and hygrometer and a wearable electrical watch. **d** CV curves at a scan rate of 50 mV s^−1^ and **e** capacitance retention (the inset is the galvanostatic discharge curves at a current density of 5 A g^−1^) of single wire supercapacitor when subjected to various bending conditions. **f**–**i** Digital camera images of two wire-type supercapacitors connected in series at different bending conditions: **f** flat, **g** bent at > 150°, **h** crumpled, and **i** recovered condition

To further consider the performance of the fabricated supercapacitors in real-world applications, we connected wire supercapacitors either in series or in parallel, to boost the ability of the supercapacitor for powering various portable electronic devices (The electrochemical data are shown in Fig. S31). The device efficiently powered 5-mm-diameter green (3.5 V, 20 mA), white (3.5 V, 20 mA), and red (2.3 V, 20 mA) round light-emitting diode (LED) indicators after being charged for tens of seconds at 3.6 V, as shown in Fig. 4a. In addition, the red LED remained very bright after 30 min and was even able to operate as an indicator after 80 min (Movie S1 and Fig. S22). Figure 4b shows the LCD devices operating as indicators for temperature, time, alarm, and stop watch, which verifies that our devices are operating perfectly. The flexible supercapacitor also successfully powered an electrical watch and a thermometer and hygrometer, as shown in Fig. 4c, suggesting the strong potential of our supercapacitor in any portable and wearable device.

In order to demonstrate the flexibility of our device for smart wearable and implantable applications, we obtained the CV curves of the hybrid supercapacitors at 50 mV s^−1^ with a bending angle from 0° to 150°, as shown in Figs. 4d and S23. The shape of the CV curves and FE-SEM images (Fig. S24) with various bending angles reveals that there is no significant difference, indicating the excellent mechanical stability for flexible energy storage system. When the flat wire supercapacitor device was severely bent at over 150°, crumpled, and recovered back to 0° in sequence, over 88% capacitance of the recovered device was retained as shown in Figs. 4e and S25, S26. Furthermore, we measured charge/discharge profiles in the device with/without bending conditions and calculated coulombic efficiency as a function of the current density as shown in Fig. S27. As a demonstration of the practical potential of our flexible supercapacitor, we powered green LED lights by the hybrid supercapacitors under various deformation conditions, as shown in Fig. 4f-i. All the devices successfully lit the LED, and the light was still bright when the device was returned to the initial condition, revealing that deformation had nearly no effect on the high performance of the flexible hybrid supercapacitor. These results prove the superior electrochemical performance and mechanical endurance of the compact hybrid supercapacitor under various deformation conditions, attributed to the robust 3D-interconnected nature of flexible wire-shaped devices.

## Conclusions

In summary, we have fabricated a facile flexible wire-shaped hybrid supercapacitor by the integration of NiCo LDH/3D-Ni electrode via a simple electrodeposition and hydrothermal method. Fast electron transport and easy migration of electrolyte ions in the solid electrolyte, which are critical for the high-performance flexible devices with a compact electrode, were indeed achieved by the deposition of dendritic active materials on the 3D-Ni/Ni network with abundant accessible active sites in a porous structure, leading to the outstanding electrochemical properties of the devices. The interconnected and compact electrode delivered a high volumetric capacitance of 73 F cm^−3^ (2446 F g^−1^), excellent rate capability, and cycle stability. In particular, the hybrid supercapacitor exhibited a battery-like energy density of 153.3 Wh kg^−1^ at a power density of 8810 W kg^−1^. Furthermore, it is successfully performed as a flexible energy storage device for a green LED light and a wearable watch under various bending conditions. These promising results demonstrate that the 3D-interconnected electrode in the flexible wire-shaped supercapacitor has remarkable potential in a wide range of flexible, wearable, and portable electronic device applications.

## Electronic supplementary material

Below is the link to the electronic supplementary material.

Supplementary material 1 (AVI 9199 kb)

Supplementary material 2 (PDF 2467 kb)
