# Supplementary material for: Wire-Shaped 3D-Hybrid Supercapacitors as Substitutes for Batteries
Source: Nanomicro Lett. 2020 Jan 20;12:28. doi: 10.1007/s40820-019-0356-z (PMC7770839; doi:10.1007/s40820-019-0356-z)
Supplement: Supplementary file 2 — Supplementary material 2 (PDF 2467 kb) [file 40820_2019_356_MOESM2_ESM.pdf]

Supporting Information for

Wire-Shaped 3D-Hybrid Supercapacitors as Substitutes for Batteries

Kyeong-Nam Kang†[1], Ananthakumar Ramadoss†[2], Jin-Wook Min[1], Jong-Chul Yoon[1], Deokjung Lee[3], Seok Ju Kang[1], Ji-Hyun Jang[1,*]

[1]School of Energy and Chemical Engineering, Low Dimensional Carbon Materials Center, Ulsan National Institute of Science and Technology, Ulsan 44919, Republic of Korea

[2]Laboratory for Advanced Research in Polymeric Materials, Central Institute of Plastic Engineering and Technology, Bhubaneswar 751024, India

[3]School of Mechanical, Aerospace and Nuclear Engineering, Ulsan National Institute of Science and Technology, Ulsan 44919, Republic of Korea

†Kyeong-Nam Kang and Ananthakumar Ramadoss contributed equally to this work

*Corresponding author. E-mail: clau@unist.ac.kr (Ji-Hyun Jang)

S1 Calculation Methods

S1.1 Equations

The optimum mass ratio of positive electrode to negative electrode was calculated by Eq. S1:

$$m_+ / m_- = V_- C_- / V_+ C_+ \qquad \text{(S1)}$$

where m is the mass of electroactive materials, V is the potential window and C represents the specific capacitance, respectively.

The gravimetric, volumetric, areal and length capacitance of NiCo LDH/3D-Ni nanostructures electrode materials were estimated from the cyclic voltammetry and galvanostatic charge/discharge profiles using Eqs. S2-S7:

Cyclic Voltammetry:

Capacitance: $$C = \frac{\int i dV}{2\nu \Delta V} \qquad \text{(S2)}$$

Galvanostatic charge/discharge:

Capacitance: $$C = \frac{I \times \Delta t}{\Delta V} \qquad \text{(S3)}$$

Gravimetric capacitance: $$C_g = \frac{C}{m} \qquad \text{(S4)}$$

Volumetric capacitance: $$C_V = \frac{C}{V} \qquad \text{(S5)}$$

Areal capacitance: $$C_A = \frac{C}{A} \qquad \text{(S6)}$$

Length capacitance: $$C_l = \frac{C}{l} \qquad \text{(S7)}$$

Where C is the capacitance (F), C_g is the gravimetric capacitance (F g^{-1}), C_v is the volumetric capacitance (F cm^{-3}), C_a is the areal capacitance (F cm^{-2}), C_l is the length capacitance (F cm^{-1}), ν is the sweep rate (mV s^{-1}), $\triangle V$ is the potential window (V), I is the discharge current (A), $\triangle t$ is the discharge time (s), v is the volume of the electrode material (cm^3), m is the mass of the active material (g), A is the area of the electroactive material (cm^2), l is the length of the electrode material and $\int idV$ is the integral area of the CV curve (A).

- Length of the active electrode: 3.75 cm
- Mass of the active materials: 0.29 mg
- Radius of the 3D-NiCo-LDH@Ni: 0.02875 cm
- Area of the electrode: $2\pi rl + 2\pi r^2$

$= 2*3.14*0.02875*3.75+ [2*3.14*(0.02875)^2]$

$=0.682253\ cm^2$

- Volume of the electrode: $\pi r^2 l$

$=3.14*(0.02875)^2*3.75$

$=9.73275*10^{-3}\ cm^3$

S1.2 Calculating the Gravimetric Capacitance of NiCo LDH/3D-Ni, Mn_3O_4/3D-Ni, and NiCo LDH/3D-Ni//Mn_3O_4/3D-Ni Electrodes [S1]

$$C_s = \frac{i}{m\,(\Delta V/\Delta t)}$$

- ✓ C → specific capacitance
- ✓ m → mass of active material
- ✓ ΔV → window range
- ✓ Δt → discharge time
- ✓ i → current density

The NiCo LDH/3D-Ni electrode:

$$C= \frac{(0.5\times10^{-3}\ A)}{(0.29\times10^{-3}\ g)(0.5\ V - (-0.2\ V))/(948.9\ s)} = \frac{1.72\ A/g}{(0.7\ V)/(948.9\ s)} = 2337.4\ F/g$$

$$C= \frac{(1\times10^{-3}\ A)}{(0.29\times10^{-3}\ g)\times(0.5\ V- (-0.2\ V))/(447.0\ s)} = \frac{3.45\ A/g}{(0.7\ V)/(447.0\ s)} = 2202.0\ F/g$$

$$C= \frac{(2.5\times10^{-3}\ A)}{(0.29\times10^{-3}\ g)\times(0.5\ V - (-0.2\ V))/(173.0\ s)} = \frac{8.62\ A/g}{(0.7\ V)/(173.0\ s)} = 2130.5\ F/g$$

$$C= \frac{(5\times10^{-3}\ A)}{(0.29\times10^{-3}\ g)\times(0.5\ V - (-0.2\ V))/(82.0\ s)} = \frac{17.24\ A/g}{(0.7\ V)/(82.0\ s)} = 2019.7\ F/g$$

$$C= \frac{(7.5\times10^{-3}\ A)}{(0.29\times10^{-3}\ g)\times(0.5\ V - (-0.2\ V))/(53.0\ s)} = \frac{25.86\ A/g}{(0.7\ V)/(53.0\ s)} = 1958.1\ F/g$$

$$C=\frac{(10\times10^{-3}\ A)}{(0.29\times10^{-3}\ g)\times(0.5\ V-(-0.2\ V))/(39.0\ s)}=\frac{34.48\ A/g}{(0.7\ V)/(39.0\ s)}=1921.2\ F/g$$

$$C=\frac{(15\times10^{-3}\ A)}{(0.29\times10^{-3}\ g)\times(0.5\ V-(-0.2\ V))/(25.0\ s)}=\frac{51.72\ A/g}{(0.7\ V)/(25.0\ s)}=1847.3\ F/g$$

$$C=\frac{(20\times10^{-3}\ A)}{(0.29\times10^{-3}\ g)\times(0.5\ V-(-0.2\ V))/(18.0\ s)}=\frac{68.97\ A/g}{(0.7\ V)/(18.0\ s)}=1773.4\ F/g$$

The Mn_3O_4/3D-Ni electrode:

$$C=\frac{(2\times10^{-3}\ A)}{(2\times10^{-3}\ g)(0.8\ V)/(675.5\ s)}=\frac{1.0\ A/g}{(0.8\ V)/(675.5\ s)}=848.8\ F/g$$

$$C=\frac{(5\times10^{-3}\ A)}{(2\times10^{-3}\ g)\times(0.8\ V)/(148.2\ s)}=\frac{2.5\ A/g}{(0.8\ V)/(148.2\ s)}=463.1\ F/g$$

$$C=\frac{(10\times10^{-3}\ A)}{(2\times10^{-3}\ g)\times(0.8\ V)/(53.8\ s)}=\frac{5\ A/g}{(0.8\ V)/(53.8\ s)}=336.3\ F/g$$

$$C=\frac{(20\times10^{-3}\ A)}{(2\times10^{-3}\ g)\times(0.8\ V)/(24.6\ s)}=\frac{10\ A/g}{(0.8\ V)/(24.6s)}=307.5\ F/g$$

The NiCo LDH/3D-Ni //Mn_3O_4/3D-Ni device:

$$C=\frac{(0.45\times10^{-3}\ A)}{(0.9\times10^{-3}\ g)\times(1.77\ V)/(1172.3\ s)}=\frac{0.5\ A/g}{(1.77\ V)/(1172.3\ s)}=331.0\ F/g$$

$$C=\frac{(0.9\times10^{-3}\ A)}{(0.9\times10^{-3}\ g)\times(1.77\ V)/(606.8\ s)}=\frac{1\ A/g}{(1.77\ V)/(606.8\ s)}=342.5\ F/g$$

$$C=\frac{(2.25\times10^{-3}\ A)}{(0.9\times10^{-3}\ g)\times(1.79\ V)/(246.6\ s)}=\frac{2.5\ A/g}{(1.79\ V)/(246.6\ s)}=344.25\ F/g$$

$$C=\frac{(4.5\times10^{-3}\ A)}{(0.9\times10^{-3}\ g)\times(1.79\ V)/(109.7\ s)}=\frac{5\ A/g}{(1.79)/(109.7\ s)}=307.25\ F/g$$

$$C=\frac{(6.75\times10^{-3}\ A)}{(0.9\times10^{-3}\ g)\times(1.77\ V)/(58.6\ s)}=\frac{7.5\ A/g}{(1.77\ V)/(58.6\ s)}=248.0\ F/g$$

$$C=\frac{(9\times10^{-3}\ A)}{(0.9\times10^{-3}\ g)\times(1.77\ V)/(38.1\ s)}=\frac{10\ A/g}{(1.77\ V)/(38.1\ s)}=215.0\ F/g$$

S1.3 Calculating the Energy Density and Power Density of the Hybrid Supercapacitors

$$E=\frac{1}{2}C_tV^2 \qquad (S8)$$

$$P=\frac{E}{t} \qquad (S9)$$

where C_t is the specific capacitance of the supercapacitor based on the total mass of the two electrodes (F g^{-1}), V is the potential window in the discharge process, and t is the discharge time (s).

The calculated capacitance of the hybrid supercapacitor was,

$$C_t = \frac{(0.5\times10^{-3}\ \mathrm{A})}{(1.0\times10^{-3}\ \mathrm{g})(1.8-0.0)/(1172.3\ \mathrm{s})} = \frac{0.5\ A/g}{(1.8\text{-}0.0)/(1172.3.0\ \mathrm{s})} = 331.0\ F/g$$

$$\mathrm{E} = \frac{1}{2}(331.0)(1.8)^2\left(\frac{1000}{3600}\right) = 144.2\frac{Wh}{kg},\ \ P = \frac{144.2}{1172.3}(3600) = 442.7\ \frac{W}{kg}$$

$$C_t = \frac{(1.0\times10^{-3}\ \mathrm{A})}{(1.0\times10^{-3}\ \mathrm{g})(1.77-0.0)/(606.8\ \mathrm{s})} = \frac{1\ A/g}{(1.8\text{-}0.0)/(606.8\ \mathrm{s})} = 342.5\ F/g$$

$$\mathrm{E} = \frac{1}{2}(342.5)(1.8)^2\left(\frac{1000}{3600}\right) = 149.3\frac{Wh}{kg},\ \ P = \frac{149.3}{606.8}(3600) = 885.9\ \frac{W}{kg}$$

$$C_t = \frac{(2.6\times10^{-3}\ \mathrm{A})}{(1.0\times10^{-3}\ \mathrm{g})(1.8-0.0)/(246.6\ \mathrm{s})} = \frac{2.5\ A/g}{(1.8\text{-}0.0)/(246.6\ \mathrm{s})} = 344.3\ F/g$$

$$\mathrm{E} = \frac{1}{2}(344.3)(1.8)^2\left(\frac{1000}{3600}\right) = 153.3\frac{Wh}{kg},\ \ P = \frac{153.3}{246.6}(3600) = 2238.5\ \frac{W}{kg}$$

$$C_t = \frac{(5.2\times10^{-3}\ \mathrm{A})}{(1.0\times10^{-3}\ \mathrm{g})(1.8-0.0)/(109.6\ \mathrm{s})} = \frac{5\ A/g}{(1.8\text{-}0.0)/(109.6\ \mathrm{s})} = 307.2\ F/g$$

$$\mathrm{E} = \frac{1}{2}(307.2)(1.8)^2\left(\frac{1000}{3600}\right) = 135.8\frac{Wh}{kg},\ \ P = \frac{135.8}{109.6}(3600) = 4459\ \frac{W}{kg}$$

$$C_t = \frac{(7.8\times10^{-3}\ \mathrm{A})}{(1.0\times10^{-3}\ \mathrm{g})(1.8-0.0)/(58.6\ \mathrm{s})} = \frac{7.5\ A/g}{(1.8\text{-}0.0)/(58.6\ \mathrm{s})} = 248.0\ F/g$$

$$\mathrm{E} = \frac{1}{2}(248.0)(1.8)^2\left(\frac{1000}{3600}\right) = 108.2\frac{Wh}{kg},\ \ P = \frac{108.2}{58.6}(3600) = 6645.8\ \frac{W}{kg}$$

$$C_t = \frac{(10.4\times10^{-3}\ \mathrm{A})}{(1.0\times10^{-3}\ \mathrm{g})(1.8-0.0)/(37.9\ \mathrm{s})} = \frac{10\ A/g}{(1.8\text{-}0.0)/(37.9\mathrm{s})} = 215.1\ F/g$$

$$\mathrm{E} = \frac{1}{2}(215.1)(1.8)^2\left(\frac{1000}{3600}\right) = 92.8\frac{Wh}{kg},\ \ P = \frac{92.8}{37.9}(3600) = 8810.8\ \frac{W}{kg}$$

S2 Supplementary Figures

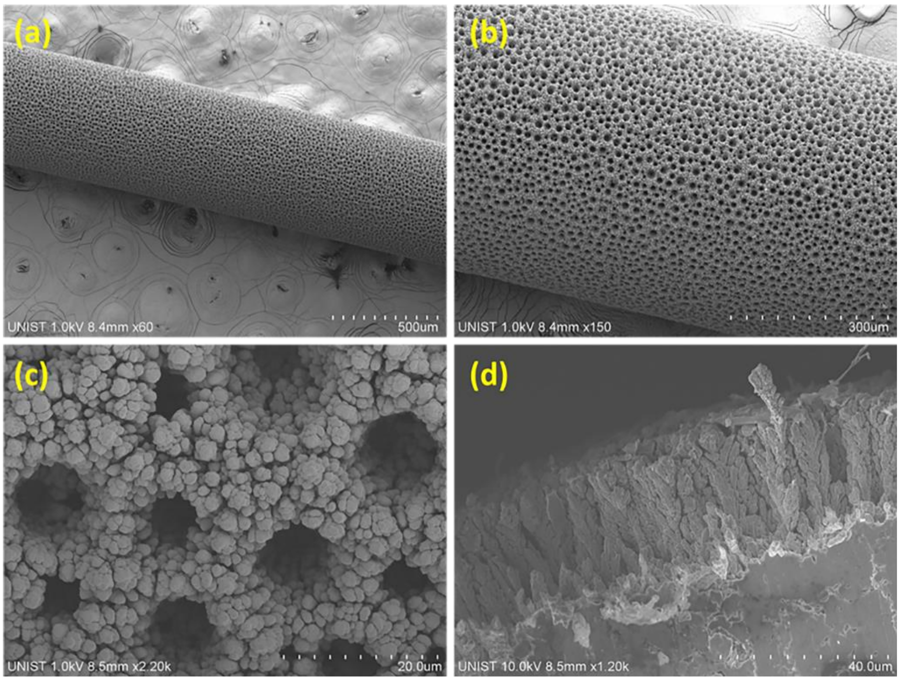

Fig. S1 Plain and cross-sectional FE-SEM images of the 3D-Ni/Ni electrode at different magnifications

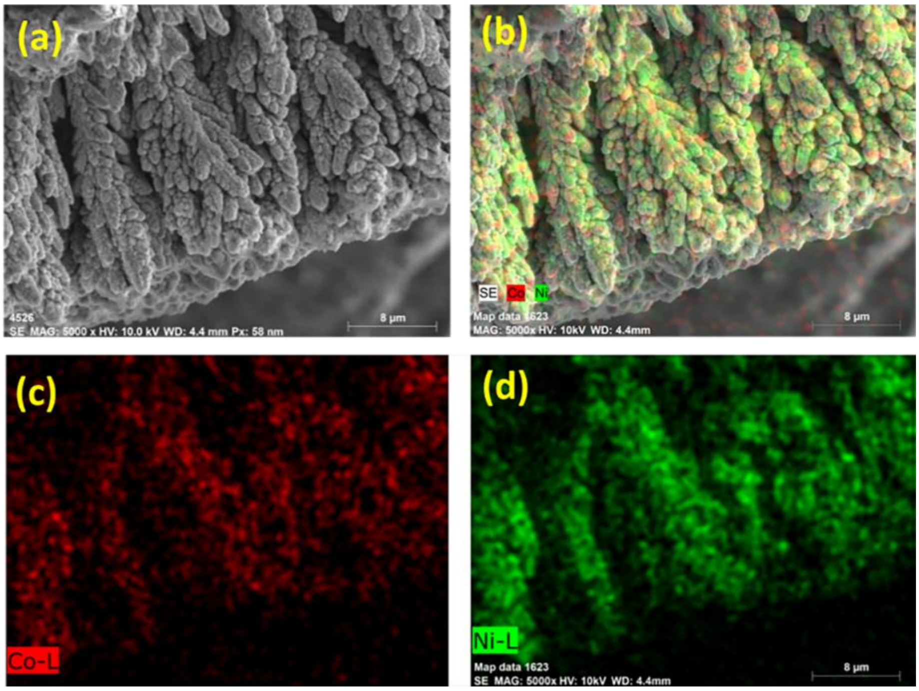

Fig. S2 Cross-sectional SEM and the corresponding EDX mapping images of NiCo-LDH/3D-Ni nanostructures

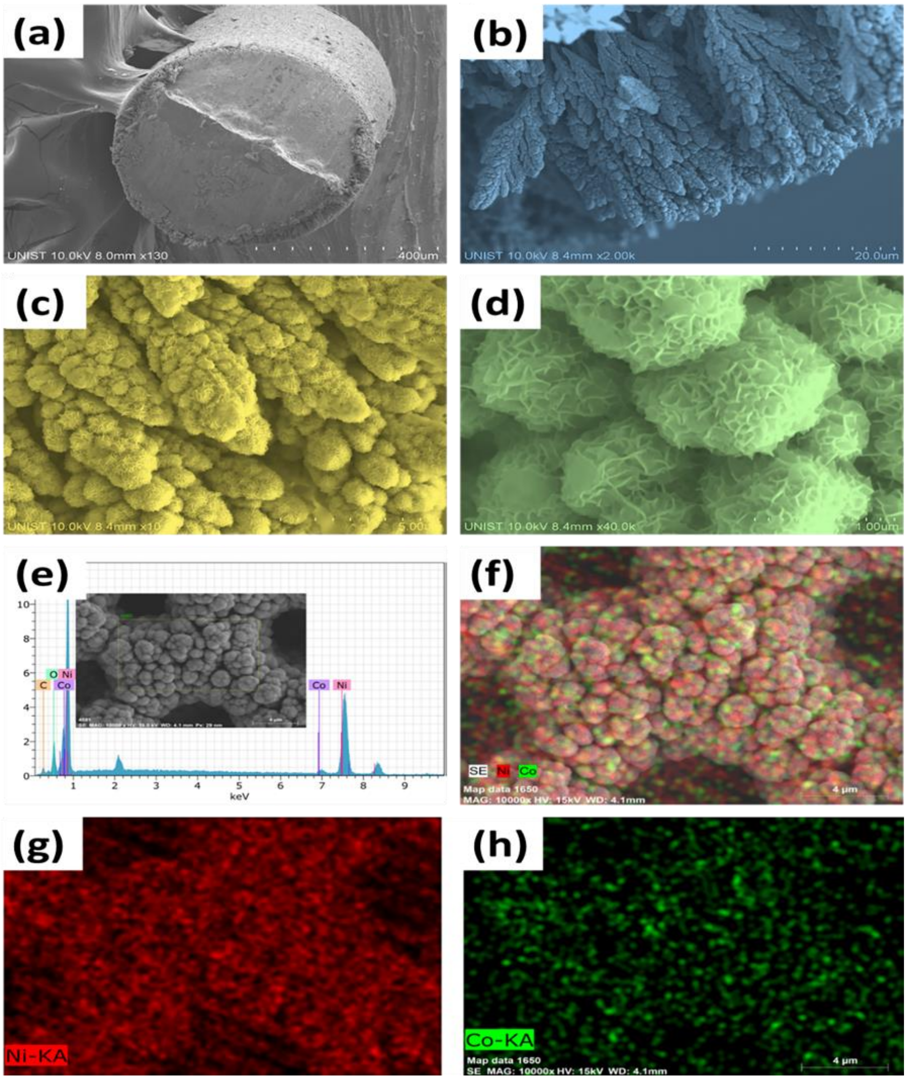

Fig. S3 (**a-d**) Cross-sectional images of NiCo LDH/3D-Ni nanostructures. EDS spectrum (**e**) and elemental mappings (**f-h**) of NiCo LDH/3D-Ni nanostructures

The EDS spectrum and elemental mapping images of 3D-NiCo LDH/Ni nanostructures are shown in Fig. S3e-h. The EDS spectrum with an inset FE-SEM image (Fig. S3e) exhibits Ni, Co, and O elemental peaks, which confirms the successful formation of NiCo LDH/3D-Ni nanostructures. From the elemental EDS mapping images in Fig. S3f-h, it was confirmed that both Ni and Co elements were uniformly distributed throughout the surface, indicating the homogenous deposition of NiCo LDHs over the entire surface of the 3D-Ni metal wire current collector.

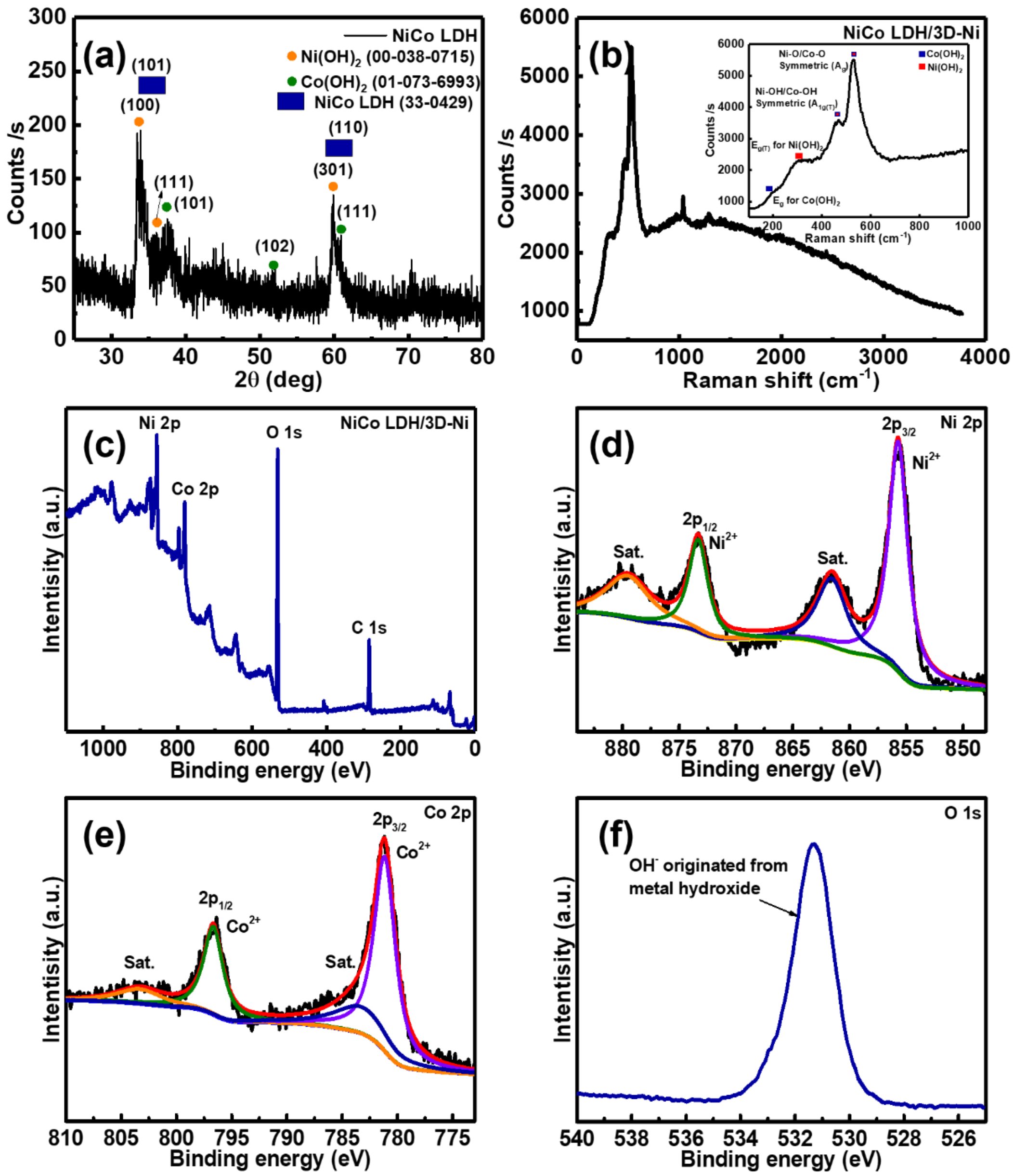

Fig. S4 (**a**) XRD spectrum of the NiCo LDH and (**b**) Raman spectrum of NiCo LDH/3D-Ni nanostructures; the inset shows the spectrum in the enlarged range between 100 cm^{-1} and 1,000 cm^{-1}. XPS spectra of NiCo LDH/3D-Ni nanostructures (**c**) survey, (**d**) Ni 2p, (**e**) Co 2p, and (**f**) O 1s

The XRD pattern of NiCo LDH is shown in Fig. S4a. All the diffraction peaks are in good agreement with the standard spectrum 1) (JCPDS No. 00-038-0715) of $Ni(OH)_2$, 2) (JCPDS No. 01-073-6993) of $Co(OH)_2$, and 3) NiCO LDH (No. 01-033-0429). The typical Raman spectrum of NiCo LDH/3D-Ni is shown in Fig. S4b. Remarkable peaks in the Raman spectrum of the NiCo LDH/3D-Ni were shown at 191, 305, 467, and 528 cm^{-1}. The peaks at 191, 467, and 528 cm^{-1} are related to the $Co(OH)_2$ phase, and the peaks at 305, 467, and 528 cm^{-1} are related to the $Ni(OH)_2$ phase. The Ni-OH/Co-OH symmetric (A1g(T)) mode and NiO-/Co-O symmetric stretching (*A*g) mode were detected at 467 and 528 cm^{-1}. The Eg(T) mode and *E*g symmetry mode for the $Ni(OH)_2$ and $Co(OH)_2$ were detected at 191 and 305 cm^{-1}, respectively. With these measurements, the NiCo

LDH active material was successfully synthesized. The elemental composition and the oxidation state of NiCo LDH/3D-Ni were evaluated by XPS, as shown in Fig. S4c-f. A typical XPS survey spectrum of NiCo LDH/3D-Ni is displayed in Fig. S4c, where nickel (Ni 2p), cobalt (Co 2p), oxygen (O 1s), and carbon (C 1s) elements were presented. The high-resolution Ni 2p spectrum in Fig. S4d exhibited two major peaks with the binding energy at 873.3 eV (Ni 2p1/2) and 855.7 eV (Ni 2p3/2) accompanied with two shakeup satellite peaks, which indicated the existence of the Ni^{2+} state. As shown in Fig. S4e, the high resolution Co 2p spectrum displayed two main peaks located at 797.2 eV (Co 2p1/2) and 781.3 eV (Co 2p3/2) accompanied by two shakeup satellites, which confirmed the cobalt is in an ionic state (Co^{2+}). Finally, the high resolution O1 peak in Fig. S4f shows a major peak at 531.3 eV, attributed to hydroxyl ions revealing the formation of metallic hydroxides [S2-S4]

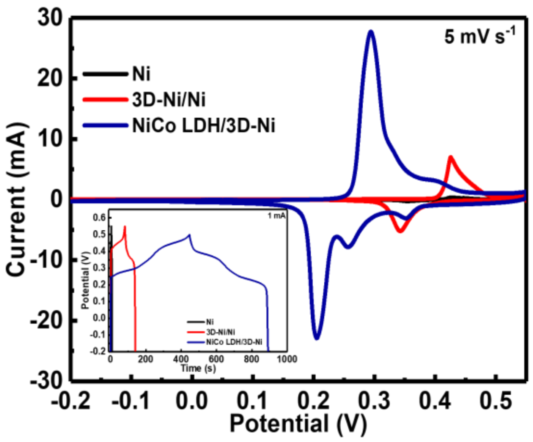

Fig. S5 Cyclic voltammetry and charge/discharge profiles of Ni, 3D-Ni, and NiCo LDH/3D-Ni at a scan rate of 5 mV s^{-1}

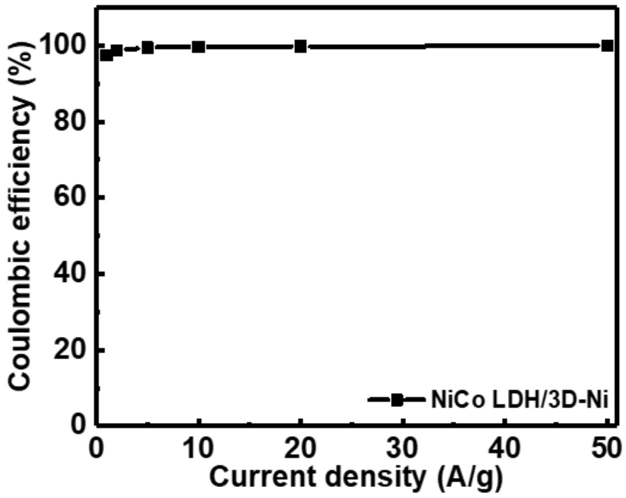

Fig S6 Coulombic efficiency of the NiCo LDH/3D-Ni electrode

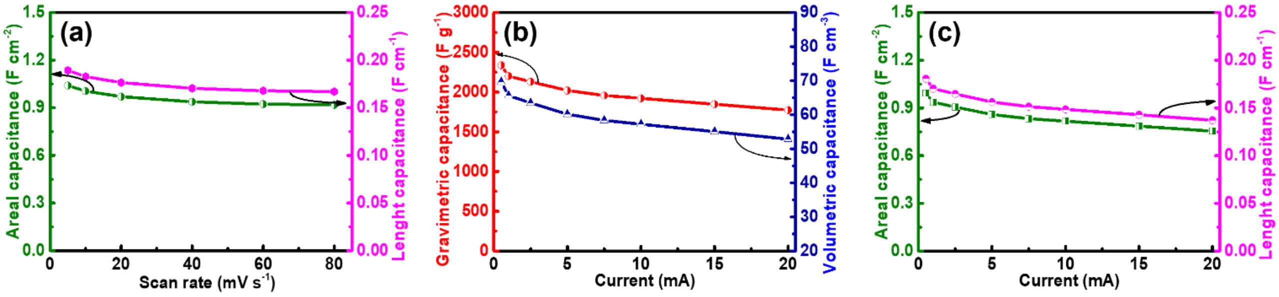

Fig. S7 (**a**) specific capacitance of NiCo LDH/3D-Ni nanostructures at various scan rates (ranging from 5 to 80 mV s^{-1}) and (**b-c**) at different currents (ranging from 0.5 to 20 mA)

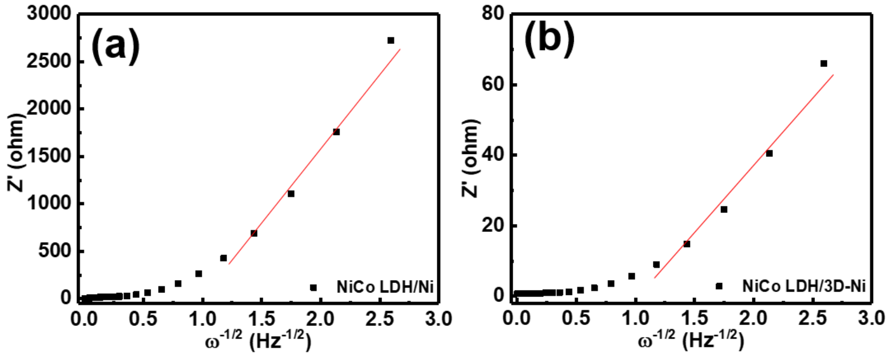

Fig. S8 plots of the real part of impedance (Z') vs. $\omega^{-1/2}$ for the (**a**) NiCo LDH/Ni and (**b**) NiCo LDH/3D-Ni electrodes

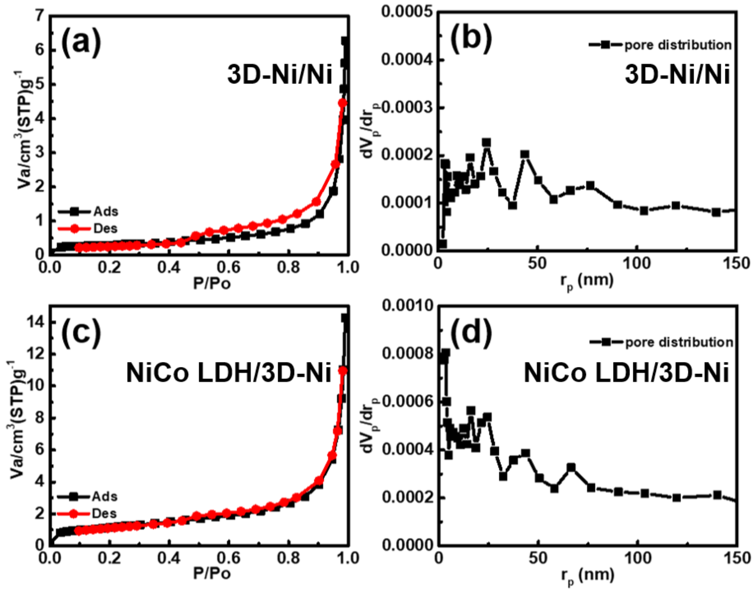

Fig. S9 BET analysis and pore size distribution of (**a-b**) 3D-Ni/Ni, and (**c-d**) NiCo LDH/3D-Ni

The nitrogen adsorption/desorption isotherms and the pore size distribution results of the two samples are shown in Fig. S8. Fig. S8a-b shows that the 3D-

Ni/Ni has a BET surface area of 0.9746 m^2 g^{-1} with meso/macro porosity, whereas the NiCo LDH/3D-Ni electrode has abundant pore distribution ranging from micropores to macropores with a 3.5215 m^2 g^{-1} BET surface area as shown in Fig. S8c-d.

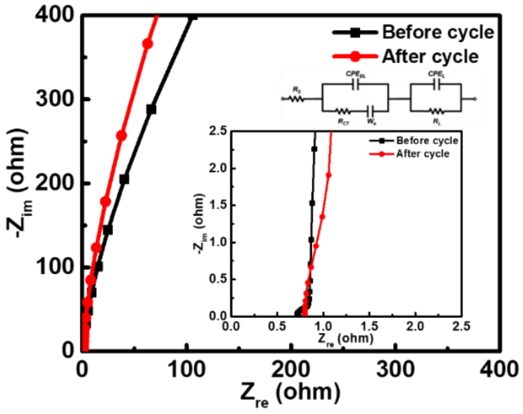

Fig. S10 Nyquist plots of NiCo LDH/3D-Ni nanostructures electrode before and after 10,000 cycles

The Nyquist plots were fitted well with the equivalent circuit model of the inset in Fig. S10. The equivalent circuit model contains several elements such as R_S, CPE_{DL}, R_{CT}, W_O, CPE_L, and R_L. R_S, the surface resistance, is the equivalent series resistance (ESR), which generally describes the resistance of the electrolyte combined with the internal resistance of the electrode. The transfer resistance (R_{CT}) demonstrates the rate of redox reactions at the electrode-electrolyte interface. The slight increase of charge transfer resistance after 10,000 cycles might be due to the loss of adhesion of some active materials from the current collector due to the continuous adsorption and desorption of OH^- ions. However, as shown in Fig. 1b-c and the Fig. S10, the overall structural stability of the active materials could be confirmed by no obvious mechanical deformation at the surface of the electrode. The Nyquist plot of 3D-NiCo LDH/Ni after 10,000 cycles exhibited a semi-circle arc at the high frequency region followed by a straight line at the low frequency region, which validates stable capacitive behavior. CPE_{DL} is the constant phase element (CPE) representing double layer capacitance, which occurs at interfaces between solids and ionic solutions due to the separation of ionic and/or electronic charges. W_O is the Warburg element, which represents the diffusion of ions into the porous electrode in the intermediate frequency region and is a result of the frequency dependence of this diffusion. R_L is the leakage resistance which is placed in parallel with CPE_L which denotes faradaic capacitance.

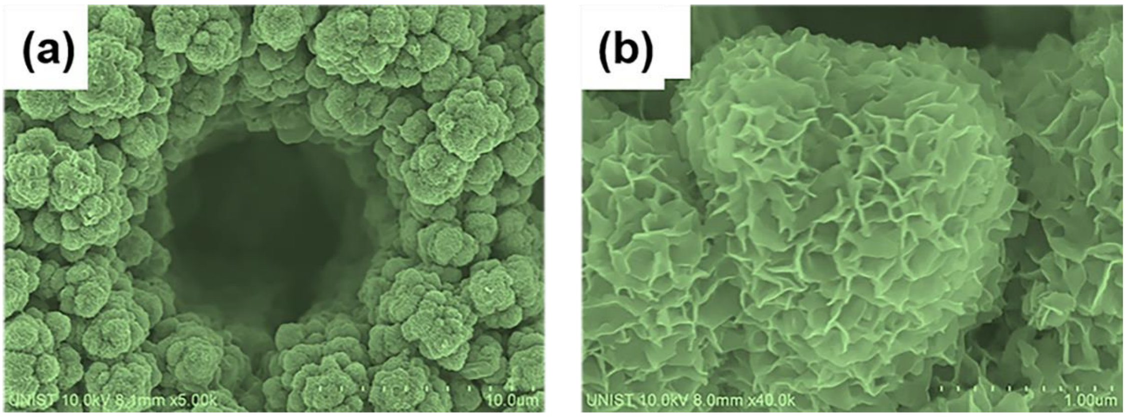

Fig. S11 (**a-b**) FE-SEM images of NiCo LDH/3D-Ni electrode after the 10,000 cycles test

Figure S11a-b present FE-SEM images of the NiCo LDH/3D-Ni nanostructure after a long-term cycling test. As shown in Fig. S11a-b, the flower-like morphology is well maintained and no obvious structural deformations are observed even after 10,000 cycles, indicating the robust nature of the NiCo LDH/3D-Ni nanostructure in hybrid supercapacitors.

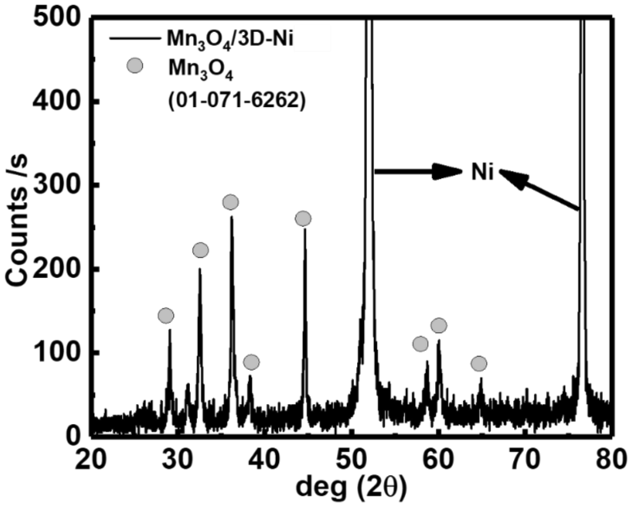

Fig. S12 XRD patterns of Mn_3O_4/3D-Ni

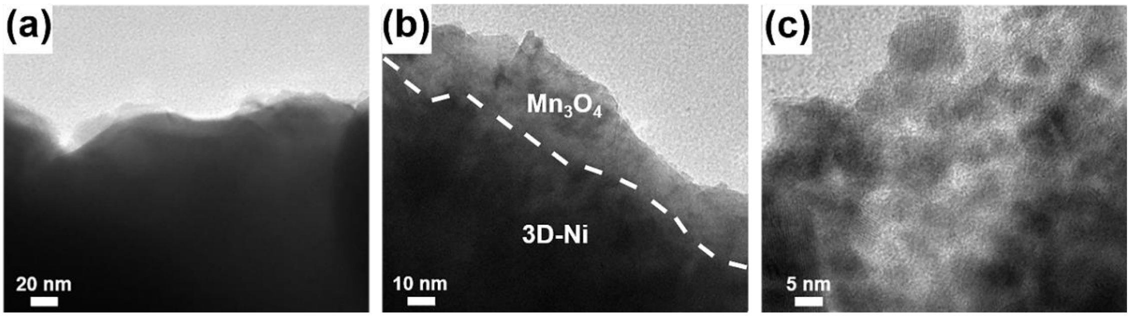

Fig. S13 TEM images of the Mn_3O_4/3D-Ni nanostructures at different magnifications

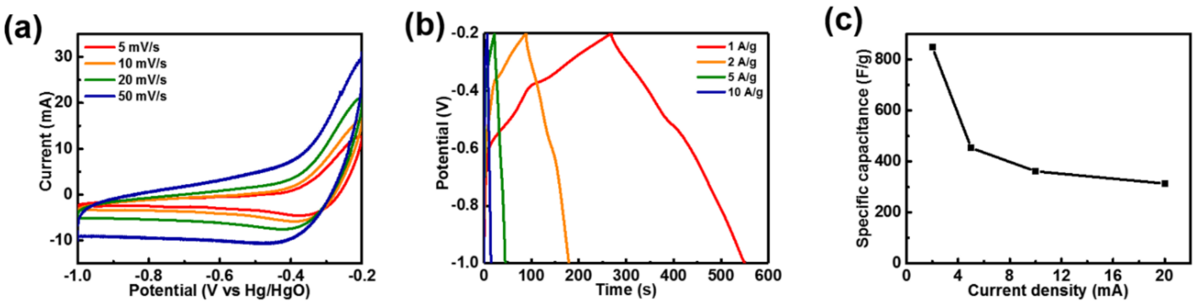

Fig. S14 Cyclic voltammetry profiles, galvanostatic charge/discharge profiles and specific capacitance of Mn_3O_4/3D-Ni nanostructures

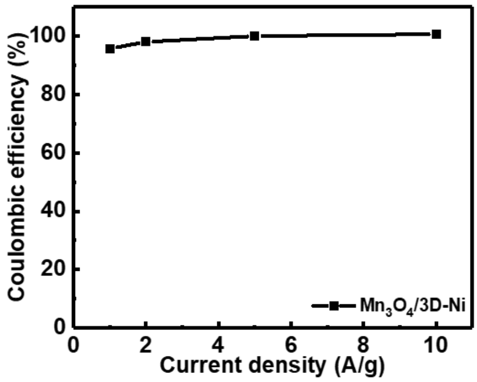

Fig. S15 Coulombic efficiency of the Mn_3O_4/3D-Ni electrode

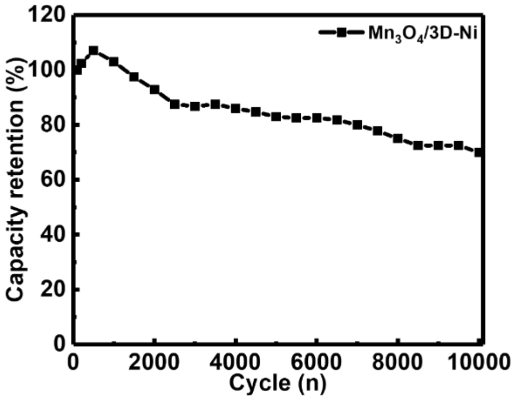

Fig. S16 Cycling performance of the Mn_3O_4/3D-Ni electrode for 10,000 cycles at a current density of 20 A g^{-1}

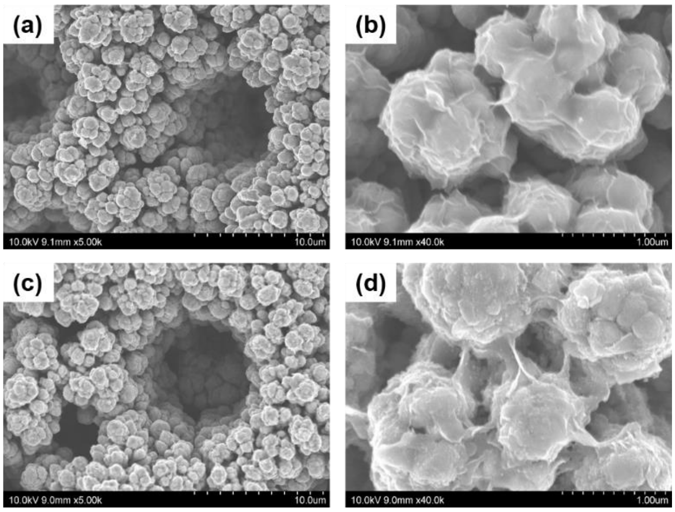

Fig. S17 FE-SEM images of the Mn_3O_4/3D-Ni electrode (**a-b**) before and (**c-d**) after the test of 10,000 cycles

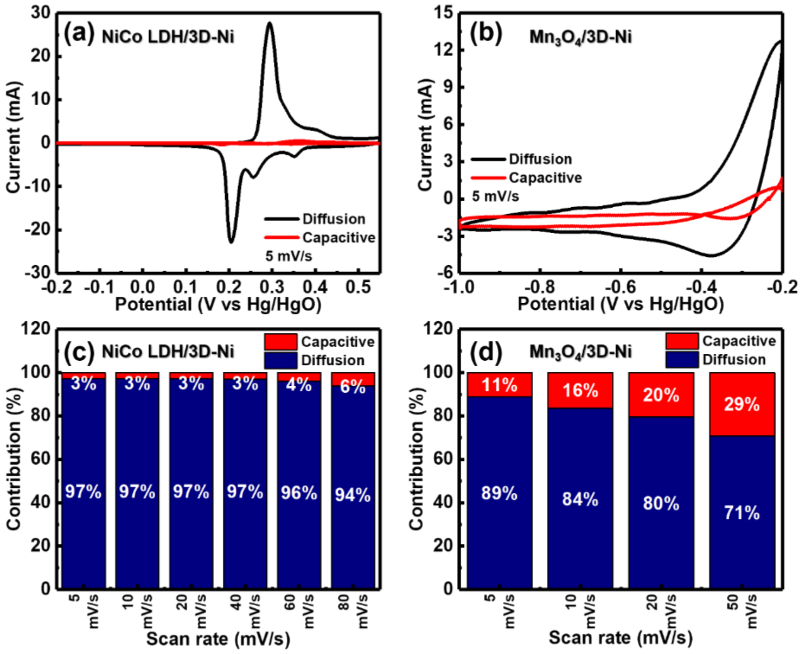

Fig. S18 Comparison of the capacitive contribution and the diffusion-controlled fraction between (**a, c**) NiCo LDH/3D-Ni and (**b, d**) Mn_3O_4/3D-Ni at different sweep rates

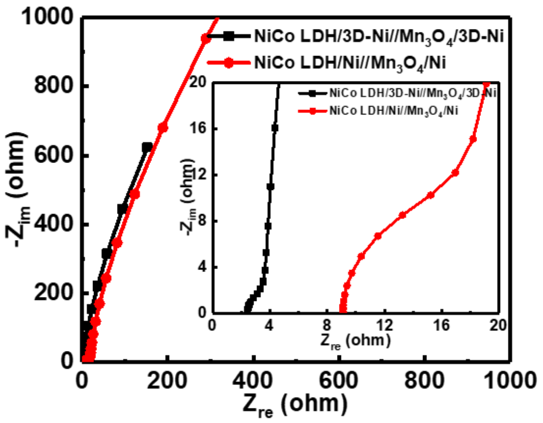

Fig. S19 Comparison of Nyquist plots of NiCo-LDH/3D-Ni//Mn_3O_4/3D-Ni and NiCo LDH/Ni//Mn_3O_4/Ni hybrid supercapacitors

Current Density (A/g)	Energy Density (Wh/kg)	Power Density (W/kg)
0.5	144.2	442.7
1	149.3	885.9
2.5	153.3	2238.5
5	135.8	4459.6
7.5	108.2	6645.8
10	92.8	8810.8

Fig. S20 Table of energy density and power density as a function of current density

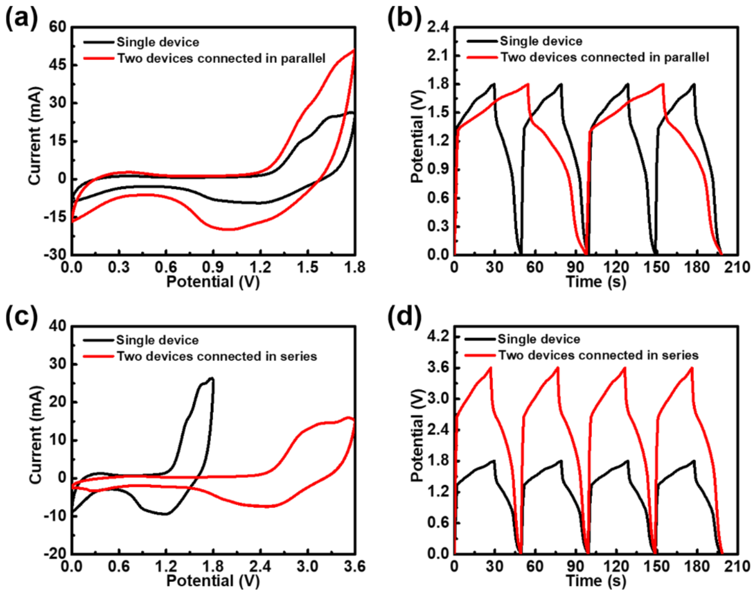

Fig. S21 CV curves of single and two wire supercapacitor devices connected in (**a**) parallel and (**c**) series. GCD curves of single and two wire supercapacitors connected in (**b**) parallel and (**d**) series at a scan rate of 20 mV s^{-1} and a current density of 20 A g^{-1}

Figure S15a-b show the CV and GCD curves of single and two wire supercapacitors connected in parallel. Compared with a single device (1.8 V), the output current and the discharge time of the two devices connected in parallel are increased by a factor of two compared with a single device at the same constant current density of 20 A g^{-1}. Fig. S15c-d show the CV and GCD curves of single and two wire supercapacitors connected in series. Compared with a single device (1.8 V), the output of the two devices connected in series exhibited a larger potential window of 3.6 V.

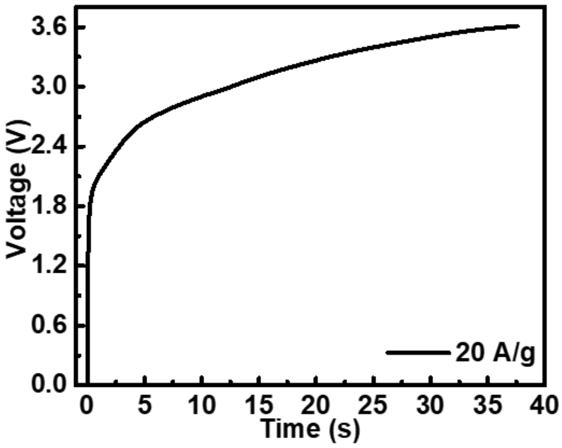

Fig. S22 Galvanostatic charging profile of the NiCo-LDH/3D-Ni//Mn_3O_4/3D-Ni hybrid supercapacitor before the LED test at a current density of 20 A g^{-1}

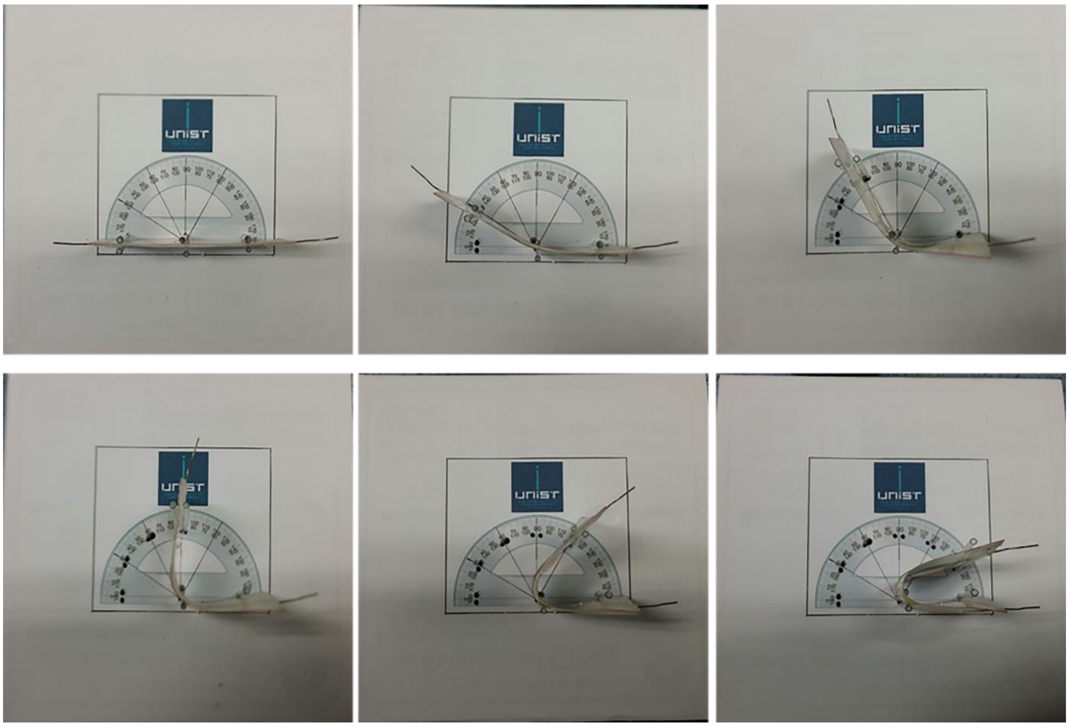

Fig. S23 Digital images of NiCo-LDH/3D-Ni//Mn_3O_4/3D-Ni as a function of bending degree

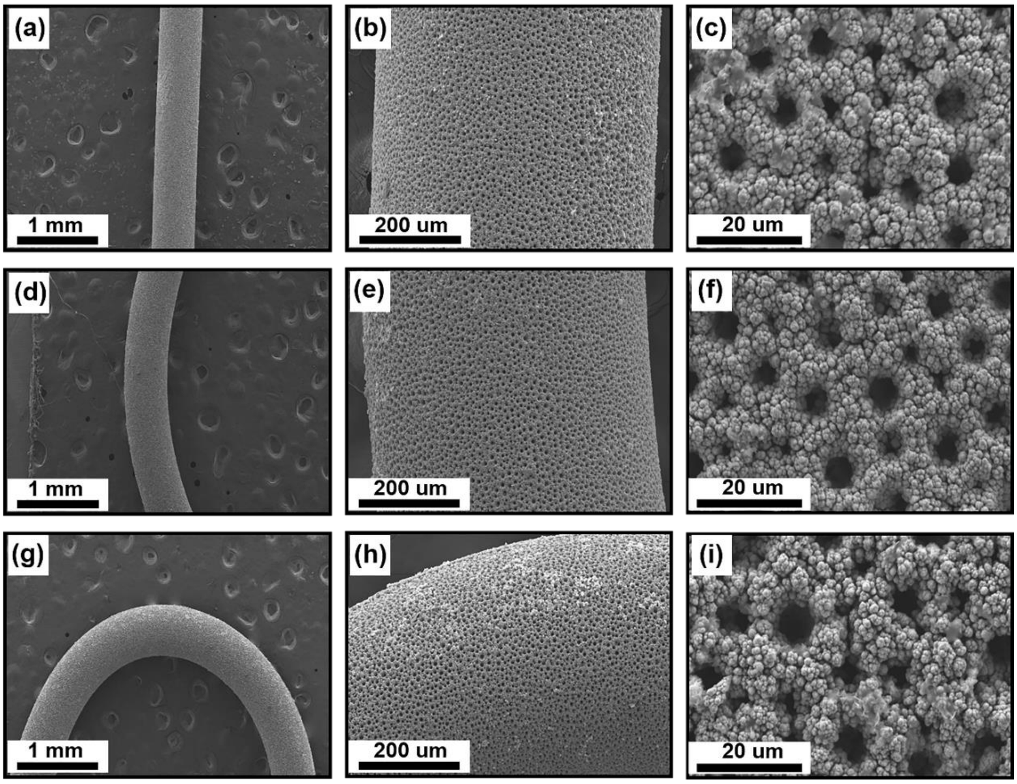

Fig. S24 FE-SEM images of NiCo LDH/3D-Ni under a bending condition: (**a-c**) 0 ^{o}C, (**d-f**) 90 ^{o}C, and (**g-i**) 180 ^{o}C

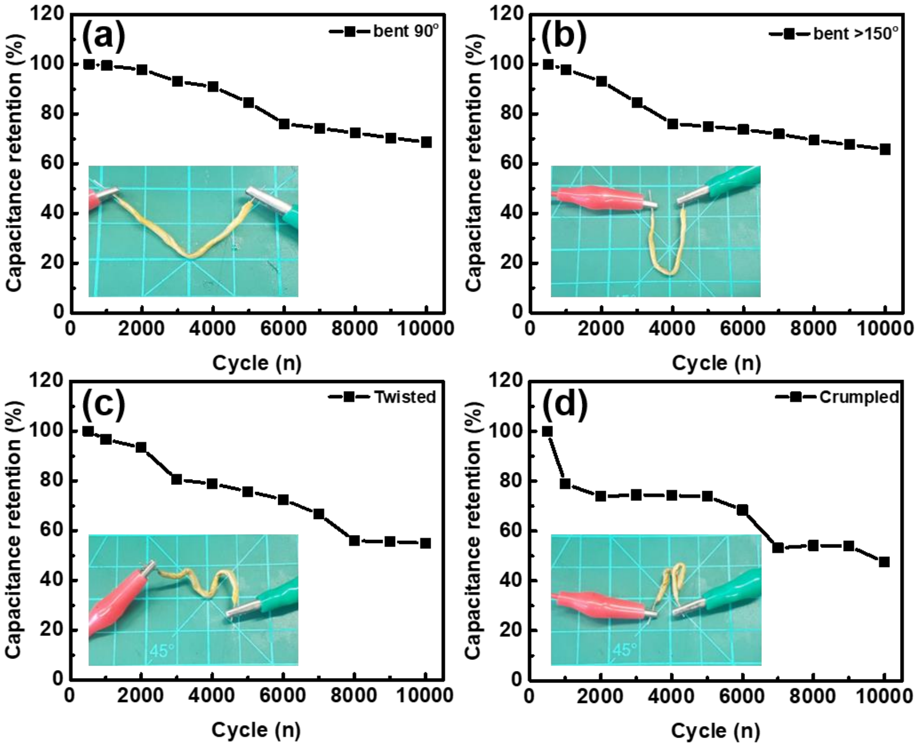

Fig. S25 Cycling performance of the hybrid supercapacitor for 10,000 cycles at a current density of 20 A g^{-1} under different deformation conditions: (**a**) bent at 90°, (**b**) bent at over 150°, (**c**) twisted, and (**d**) crumpled. Insets of the figures show photographs of each deformation condition

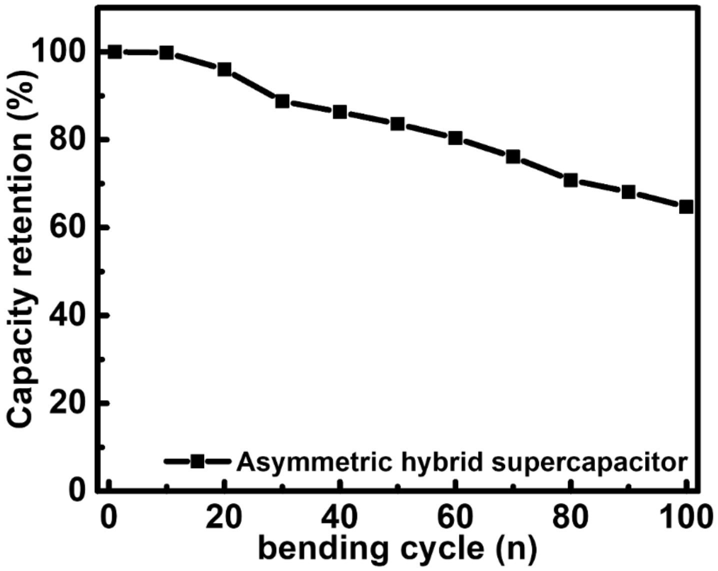

Fig. S26 Cycling performance of the hybrid supercapacitor for 100 cycles at a current density of 20 A g^{-1} under bending and recovery conditions

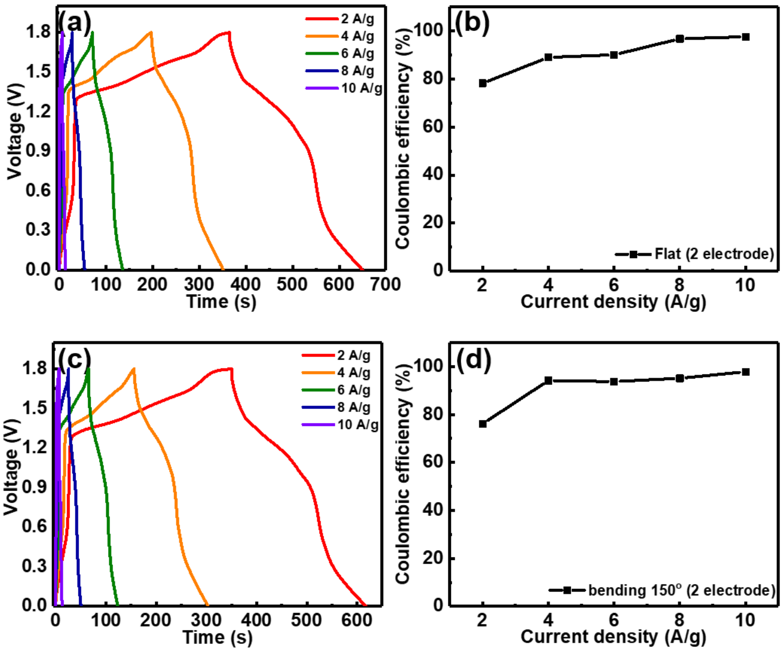

Fig. S27 Charge/discharge profiles of the NiCo LDH/3D-Ni//Mn_3O_4/3D-Ni in (**a**) flat and (**c**) over 150° bending conditions. The coulombic efficiency results as a function of the current density of the device in (**b**) flat and (**d**) over 150° bending conditions

Supplementary References

[S1]A. Laheaar, P. Przygocki, Q. Abbas, F. Beguin, Appropriate methods for evaluating the efficiency and capacitive behavior of different types of supercapacitors. Electrochem. Commun. **60**, 21-25 (2015). https://doi.org/10.1016/j.elecom.2015.07.022

[S2]J. Pu, Y. Tong, S. Wang, E. Sheng, Z. Wang, Nickel-cobalt hydroxide nanosheets arrays on Ni foam for pseudocapacitor applications. J. Powder Sources **250**, 250-256 (2014). https://doi.org/10.1016/j.jpowsour.2013.10.108

[S3]H. Peng, M. Zhou, Y. Li, X. Cui, Y. Yang, Y. Zhang, P. Xiao, Ultrahigh voltage synthesis of 2D amorphous nickel-cobalt hydroxide nanosheets on CFP for high performance energy storage device Electrochim. Acta **190**, 695-702 (2016). https://doi.org/10.1016/j.electacta.2016.01.015

[S4]J. Xing, S. Wu, K.Y. Simon Ng, Electrodeposition of ultrathin nickel-cobalt double hydroxide nanosheets on nickel foam as high-performance supercapacitor electrodes. **5**, 88780-88786 (2015). https://doi.org/10.1039/C5RA17481C

[S5]B. Vidhyadharan, I.I. Misnon, R.A. Aziz, K.P. Padmasree, M.M. Yusoff, R. Jose, Superior supercapacitive performance in electrospun copper oxide nanowire electrodes. J. Mater. Chem. A **2**, 6578 (2014). https://doi.org/10.1039/C3TA15304E

[S6]X. Wang, J. Hu, W. Liu, G. Wang, J. An, J. Lian, Ni–Zn binary system hydroxide, oxide and sulfide materials: synthesis and high supercapacitor performance. J. Mater. Chem. A **3**, 23333-23344 (2015). https://doi.org/10.1039/C5TA07169K

[S7]C. Liu, Z. Xie, W. Wang, Z. Li, Z. Zhang, Fabrication of MoOx Film as a Conductive Anode Material for Micro-Supercapacitors by Electrodeposition and Annealing. J. Electrochem. Soc. **161**, A1051-A1057 (2014). https://doi.org/10.1149/2.081406jes

[S8]J. Zhang, H. Feng, Q. Qin, G. Zhang, Y. Cui, Z. Chai, W. Zheng, Interior design of three-dimensional CuO ordered architectures with enhanced performance for supercapacitors. J. Mater. Chem. A **4**, 6357 (2016). https://doi.org/10.1039/C6TA00397D

[S9]C.-T. Hsieh, S.-M. Hsu, J.-Y. Lin, H. Teng, Electrochemical capacitors based on graphene oxide sheets using different aqueous electrolytes. J. Phys. Chem. C **115**(25), 12367-12374 (2011). https://doi.org/10.1021/jp2032687
